# Structural Distortions and Magnetic Ordering in *Ae*_2_FeO_3_Cu*Ch* (*Ae* = Ca, Sr; *Ch* = S, Se) Oxide Chalcogenides

**DOI:** 10.1021/acs.inorgchem.6c02221

**Published:** 2026-07-01

**Authors:** Robert D. Smyth, Bradley C. Sheath, Lemuel E. Crentsil, Xiaoyu Xu, Simon J. Cassidy, Maria Batuk, Pascal Manuel, Emmanuelle Suard, Andrew N. Fitch, Joke Hadermann, Simon J. Clarke

**Affiliations:** † Department of Chemistry, Inorganic Chemistry Laboratory, 6396 University of Oxford, South Parks Road, Oxford OX1 3QR, U.K.; ‡ Electron Microscopy for Materials Science (EMAT), 26660 University of Antwerp, Antwerp B-2020, Belgium; § ISIS Facility, Rutherford Appleton Laboratory Harwell Oxford, Didcot OX1 10QX, U.K.; ∥ Institut Laue-Langevin, 71 Avenue des Martyrs, CS 20156, Grenoble, Cedex 9 38042, France; ⊥ European Synchrotron Radiation Facility, 71 Avenue des Martyrs, CS 40220, Grenoble, Cedex 9 38043, France

## Abstract

The contrasting crystal and magnetic structures of four related iron oxide chalcogenides are reported. *Ae*_2_FeO_3_Cu*Ch* (*Ae* = Ca, Sr; *Ch* = S, Se) all crystallize in the Sr_2_GaO_3_CuS structure with alkaline earth iron oxide layers containing double layers of linked FeO_5_ square pyramids containing Fe^3+^ ions separated by antifluorite-type [Cu_2_ *Ch*_2_]^2–^ layers. Structural distortions occur below room temperature when the small Ca^2+^ ions are present, and these involve cooperative tilting of the FeO_5_ square pyramids. Magnetic reflections present in the diffraction patterns can be indexed using either √2*a* × √2*a* × *c* or √2*a* × √2*a* × 2*c* expansions of the nuclear cell with nearest-neighbor Fe^3+^ moments coupling antiferromagnetically and with temperature-dependent orientations relative to the crystallographic directions. The magnetic structures of these compounds are subtly different in detail, partly on account of the low directional preference of the high-spin d^5^ Fe^3+^ moments.

## Graphical abstract

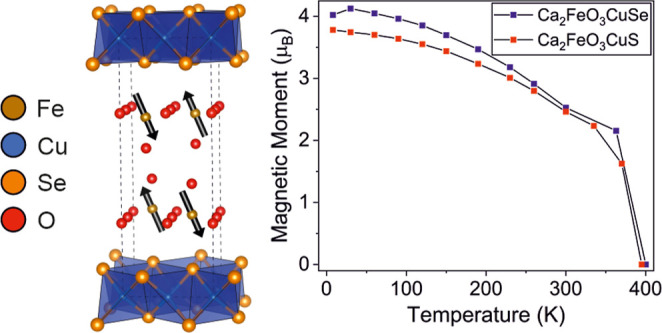

## Introduction

Materials containing multiple anions^1,2^ have recently become of great interest due to their tendency to form complex crystal structures and exhibit properties that allow them to have potential uses as superconductors,^3^ thermoelectrics,^4,5^ and battery cathodes.^6^ Because of the contrasting sizes and chemistries of the anions involved in these mixed-anion compounds, they will often segregate into alternating layers, and this anion ordering may promote ordering of transition-metal cations according to their steric and chemical requirements. This is the case for oxide chalcogenides when one transition-metal cation is more oxophilic and the other more chalcophilic. This leads to a large series of compounds with midtransition-metal oxide layers with perovskite-related structures separated by sulfide, selenide, or telluride layers containing more chalcophilic metals such as the coinage metals Cu and Ag in their monovalent states. Such compounds may exhibit complex magnetism in the oxide layers and may allow chemical tuning by topochemical processes normally operating in the chalcogenide layer, including ion exchange^7^ and chalcogenide redox processes.^8^

The inclusion of first-row transition metals within the oxide layer in oxide chalcogenides can often give rise to long-range magnetic order where the localized moments on these ions exhibit a range of magnetic ordering schemes depending on the transition metal and the oxidation state.^9–14^ The *Ae*_2_FeO_3_Cu*Ch* (*Ae* = Ca, Sr; *Ch* = S, Se) series of compounds, with Fe^3+^ cations in the oxide slab, have received some interest for their structural and magnetic properties. Sr_2_FeO_3_CuSe was explored by Berthebaud et al. in 2014 ^15^ and was found to be analogous to Sr_2_FeO_3_CuS, which was discovered by Zhu and Hor in 1997.^16^ Ca_2_FeO_3_CuSe and Ca_2_FeO_3_CuS were described by Charkin et al. in 2010,^17^ and they showed that both materials adopt the undistorted Sr_2_GaO_3_CuS structure at room temperature (RT) and have an antiferromagnetic transition present in their magnetometry data. Calculations by Suetin and Ivanovskii on Ca_2_FeO_3_CuSe and Ca_2_FeO_3_CuS predicted that the localized Fe^3+^ moments in these compounds are large in magnitude and should indeed order antiferromagnetically.^18^

Here, we examine these four compounds in detail and compare and contrast the structure and magnetic ordering in Ca_2_FeO_3_CuSe, Ca_2_FeO_3_CuS, Sr_2_FeO_3_CuSe, and Sr_2_FeO_3_CuS through the use of synchrotron X-ray diffraction, electron diffraction (ED), and variable-temperature neutron diffraction experiments.

## Experimental Section

### Synthesis

Each member of the *Ae*_2_FeO_3_Cu*Ch* (*Ae* = Ca, Sr; *Ch* = S, Se) series was synthesized on the 3 g scale under an inert atmosphere, starting from stoichiometric amounts of SrO or CaO (Alfa 99.95%), CuO (Alfa 99.995%), Fe (Alfa 99.998%), and either Se (Alfa 99.999%) or S (Alfa 99.999%). SrO was synthesized via the thermal decomposition of SrCO_3_ (Alfa 99.994%), achieved by heating SrCO_3_ under dynamic vacuum at 830 °C for 16 h and then a final treatment at 1100 °C for 4 h. The starting materials were ground together using an agate pestle and mortar until a homogeneous mixture was obtained. Two pellets of approximately 1.5 g each were then pressed under 3–4 tonnes of force in a 13 mm pellet die. The pellets were loaded into an alumina crucible, which was then sealed in an evacuated silica ampule, and these were then heated in accordance with the heating cycles in Table 1. Thorough grinding and repelletizing were carried out between the two heating cycles. The lower temperature of 400 °C used in heating cycle 1 for the sulfides was adopted in order to ensure that the sulfur reacted with the other starting materials before reaching a high vapor pressure.

**Table 1:** Heating Cycles Used in the Syntheses of *Ae*_2_FeO_3_Cu*Ch* (*Ae* = Ca, Sr; *Ch* = S, Se)

	heating cycle 1	heating cycle 2
Ca_2_FeO_3_CuSe	675 °C/12 h	675 °C/36 h
Ca_2_FeO_3_CuS	400 °C/36 h	900 °C/60 h
Sr_2_FeO_3_CuSe	900 °C/60 h	900 °C/24 h
Sr_2_FeO_3_CuS	400 °C/36 h	950 °C/60 h

### Powder Diffraction Measurements

A Bruker D8 Advance Eco diffractometer (using Cu Kα radiation and an energy-discriminating detector to suppress fluorescence from Fe) was used to obtain X-ray powder diffraction (XRPD) data in order to follow the reactions between heating steps. Higher resolution XRPD data with a much greater signal/noise ratio were collected on all four samples on the I11 beamline^19^ at the Diamond Light Source synchrotron using X-rays of wavelength ≈0.82 Å (calibrated at the start of each beam session using a Si standard) and the Multi-analyzer crystal (MAC) detector with 30 min scans. Additionally, variable temperature XRPD was carried out for each sample from 500 K down to 80 K using the Mythen-II position-sensitive detector (PSD) with temperature controlled by an Oxford Cryosystems Cryostream plus. The highest quality X-ray powder data (narrow peak widths, high Q range and high signal/noise ratio, and low absorption in transmission geometry) were collected at selected temperatures on beamline ID22 ^20^ for Ca_2_FeO_3_CuS and Ca_2_FeO_3_CuSe at the ESRF, France, using Si-calibrated X-rays of wavelength 0.35 Å and a Dectris Eiger2 × 2M-W CdTe hybrid pixel detector with cooling by an Oxford Cryosystem Cryostream 1000 plus. Measurements were collected for −10 ≤ 2θ/degrees ≤40 at 2° min^–1^ four times and then summed for a total collection time of 100 min. For all the synchrotron experiments, the samples were finely ground and contained in 0.5 mm diameter borosilicate capillaries. Neutron powder diffraction (NPD) data were obtained from both WISH^21^ (time-of-flight instrument with a white neutron beam) at ISIS, Oxford, UK, and the D2B instrument [a wavelength λ of 1.59 Å was selected using a Ge(335) monochromator] at the ILL, Grenoble, France.^22^ Neutron diffraction data over a range of temperatures below and above RT were collected from approximately 2 g of each sample loaded into 6 mm diameter thin-walled vanadium cans. The XRPD and NPD data were analyzed by Rietveld refinement using TOPAS Academic V6.^23^ Structure solution of the low temperature structures of Ca_2_FeO_3_CuS and Ca_2_FeO_3_CuSe was performed using the charge flipping algorithm in SUPERFLIP, as implemented in JANA 2020.^24^

### Transmission Electron Microscopy

Selected area electron diffraction (SAED) patterns and 3D ED series were acquired on a Tecnai G2 transmission electron microscope (TEM) operated at 200 kV and equipped with a Nanomegas DigiStar precession module. A specimen for the TEM study was prepared by mixing the material with ethanol in an ultrasonic bath and depositing a few drops of the suspension onto a copper grid covered by a holey carbon layer. For the acquisition of in-zone SAED patterns, the TEM grid was placed into a Gatan double-tilt cooling holder and cooled down to 100 K inside the microscope. For the 3D ED measurements, the TEM grid was placed into a Fischione cryo transfer tomography holder and cooled down to 100 K inside the microscope. The ED patterns were acquired from −56° to +75° with a step of 1° and an electron beam precession of 1°. The PETS2.0 software was used to analyze the data, make the 3D reconstruction of the reciprocal space, and plot the sections.^25^

### Magnetometry

The magnetic properties of each compound were measured using Quantum Design MPMS-XL or MPMS-3 SQUID magnetometers. 10–30 mg of powder was accurately weighed out into a gelatin capsule, which was secured within a plastic straw and lowered into the magnetometer. Measurements of the magnetic moments of these samples against temperature were obtained upon warming (with an applied field of 100 Oe) once the samples had been zero-field-cooled or field-cooled in the measuring field.

## Results and Discussion

### Compositions and Crystal Structures

All four target compounds were synthesized to a high degree of purity without any notable crystalline impurities evident in the synchrotron XRPD data collected on I11, as presented in Figure 1. Only in the case of Ca_2_FeO_3_CuSe were small impurities identified in data from ID22 (see Figure 8) and by ED (Figure S6). The *Ae*_2_FeO_3_Cu*Ch* (*Ae* = Ca, Sr; *Ch* = S, Se) family adopts the Sr_2_GaO_3_CuS structure type at ambient temperatures in the *P*4/*nmm* space group, as illustrated in Figure 2a. Fe^3+^ cations coordinated by a square-pyramidal arrangement of oxide anions reside within [*Ae*_2_FeO_3_]^+^ slabs, and antifluorite-type [Cu*Ch*]^−^ layers host Cu^+^ ions in tetrahedral coordination by chalcogenide anions, as illustrated in Figure 2b. The alkaline-earth sites are 8-coordinate (by 4 oxide and 4 chalcogenide ions) between the oxide and chalcogenide layers and 9-coordinate (by 9 oxide ions) within the oxide slabs.

**Figure 1:**
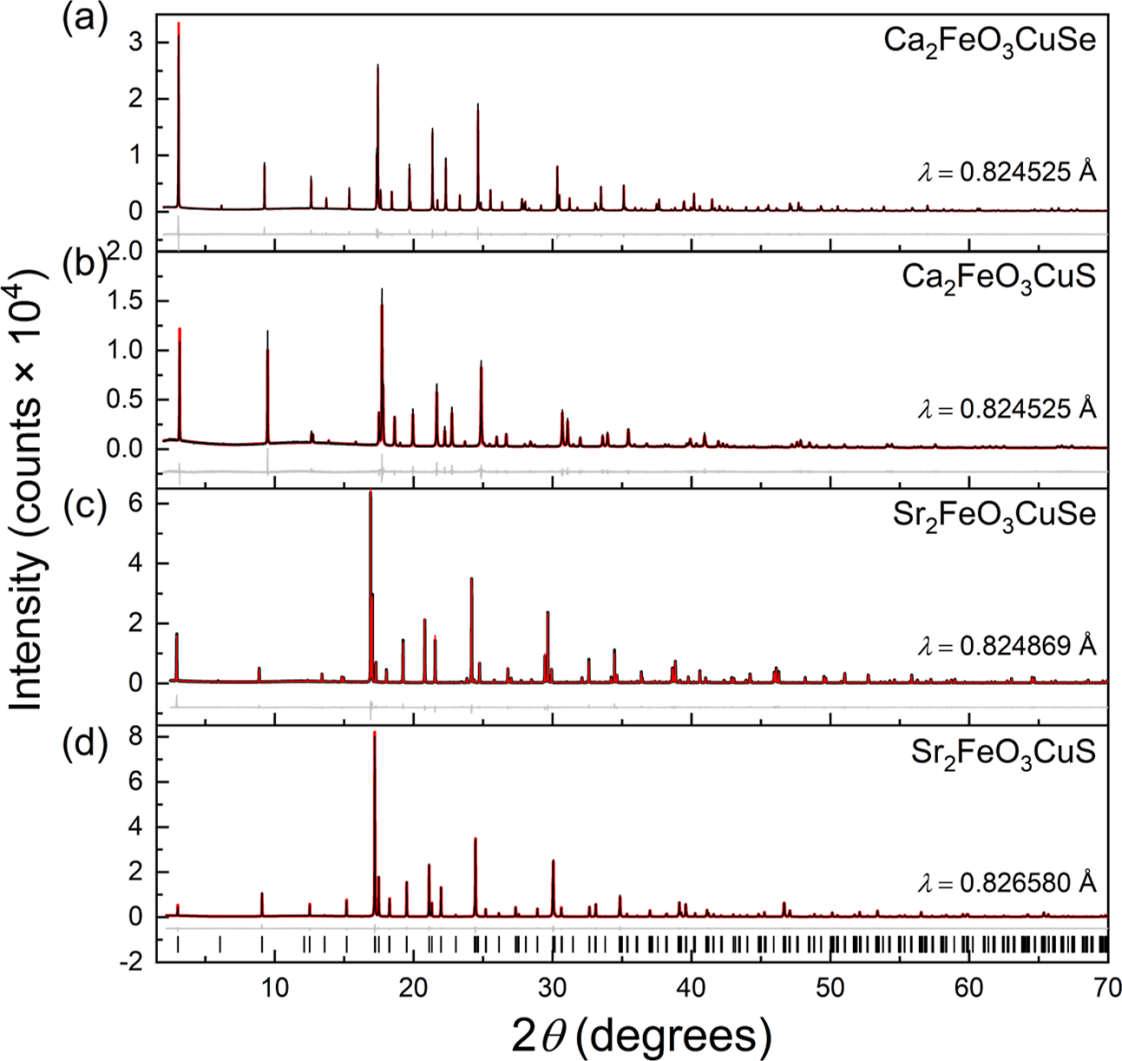
XRPD patterns measured at 300 K on I11 (MAC detector), showing the observed (black), calculated (red), and difference (gray) curves of (a) Ca_2_FeO_3_CuSe, *R*_wp_: 8.040%, (b) Ca_2_FeO_3_CuS, *R*_wp_: 8.407%, (c) Sr_2_FeO_3_CuSe, *R*_wp_: 7.007%, and (d) Sr_2_FeO_3_CuS, *R*_wp_: 5.660%.

**Figure 2:**
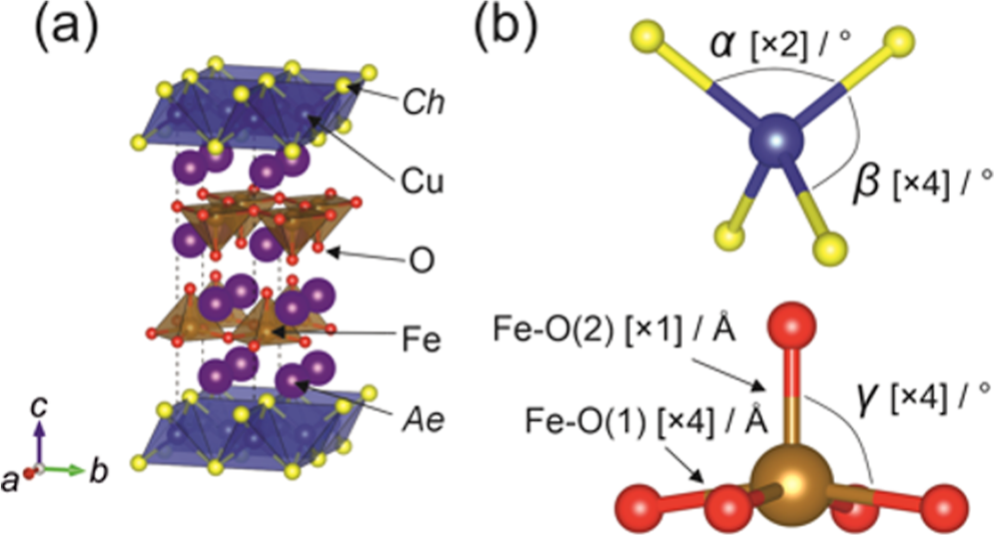
(a) The crystal structure of the *Ae*_2_FeO_3_Cu*Ch* (*Ae* = Ca, Sr; *Ch* = S, Se). (b) Definition of the α and β tetrahedral angles in the Cu*Ch* layer as well as the Fe–O(1) and Fe–O(2) bond lengths as well as the γ angle in the FeO_5_ square pyramids. The bond lengths and angles at RT are listed in Table 2.

**Figure 3:**
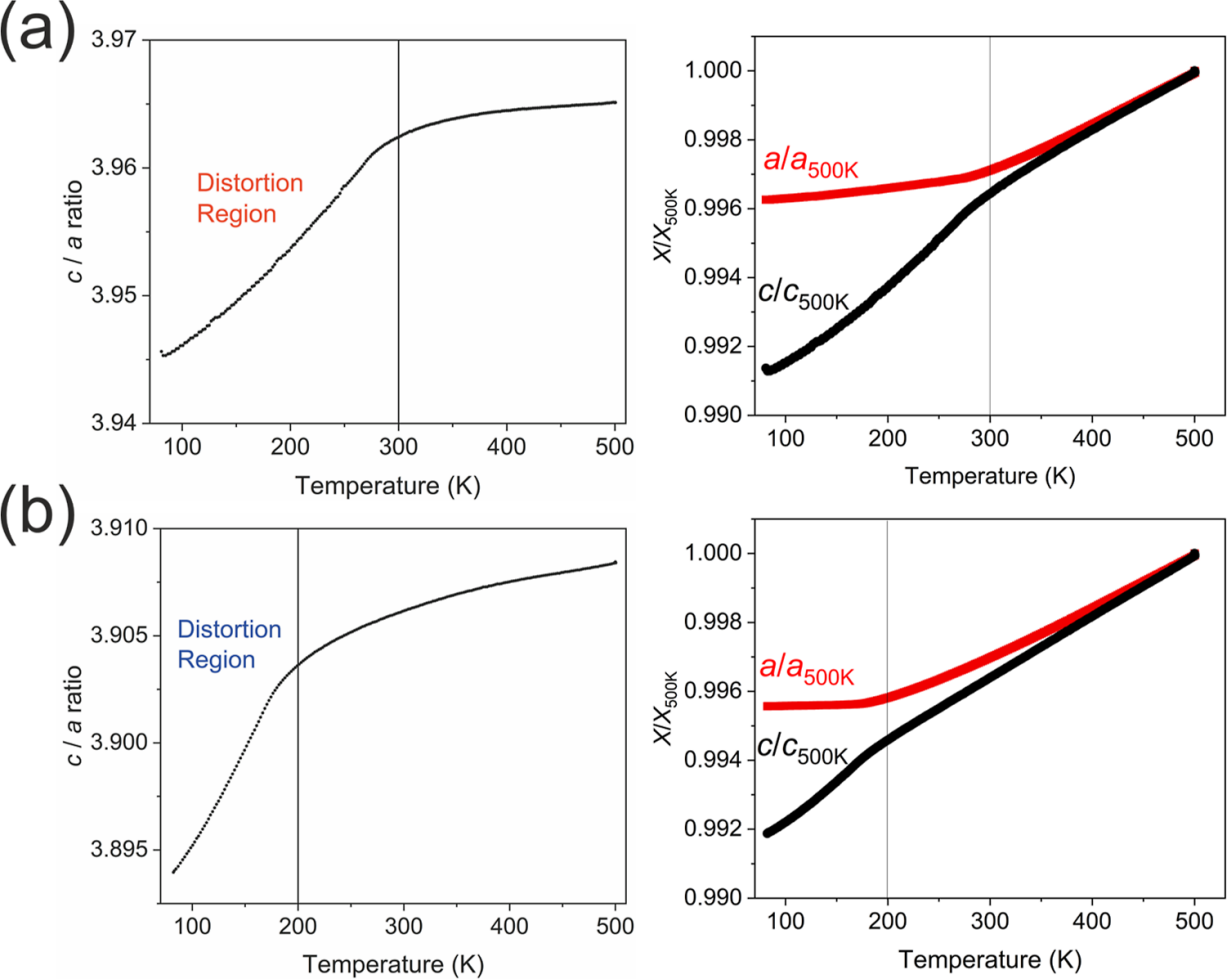
Changes in the *c*/*a* ratio and normalized *a* and *c* lattice parameters for (a) Ca_2_FeO_3_CuSe and (b) Ca_2_FeO_3_CuS; the error bars are within the data points.

**Figure 4:**
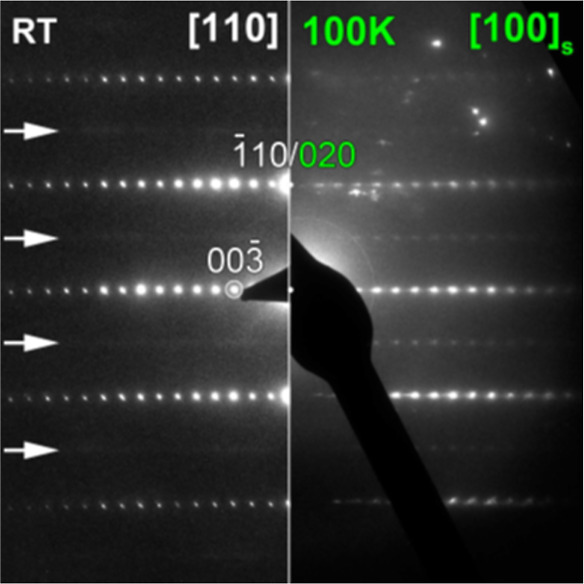
Transformation of the [110] zone of the parent cell to the [100]_s_ zone of the √2*a* × √2*a* × *c* supercell upon cooling Ca_2_FeO_3_CuSe from 300 to 100 K. The white arrows indicate the evolution of distinct diffraction spots at 100 K from weak diffuse streaks at (*h*/2 *k*/2 *l*) present at RT.

**Figure 5:**
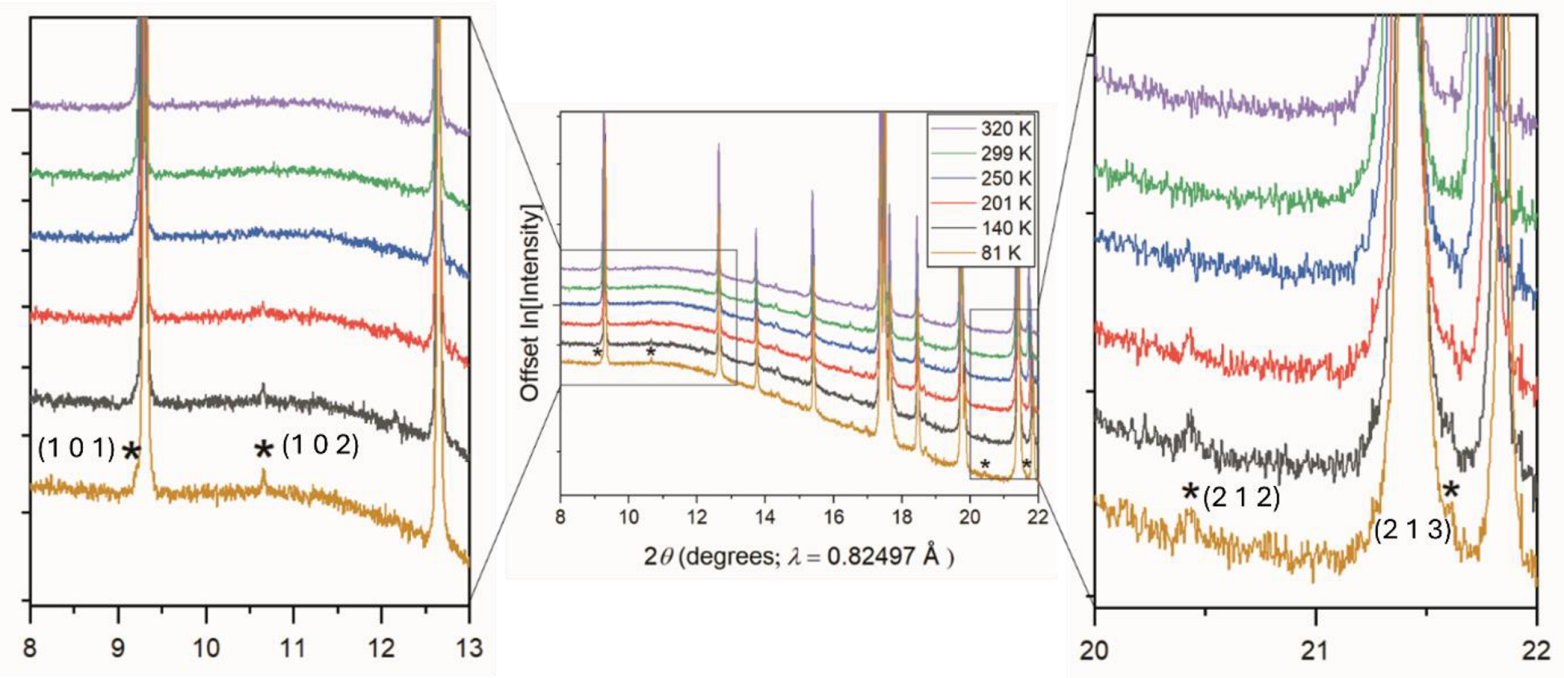
Evolution of weak supercell peaks (highlighted by an * and indices included) present in the variable temperature XRPD data of Ca_2_FeO_3_CuSe (I11, PSD detector).

**Figure 6:**
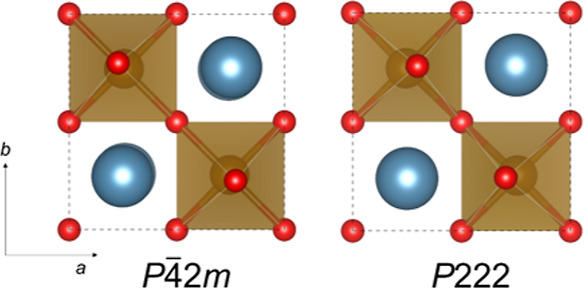
A comparison of the FeO_5_ pyramids in the *P*4̅2*m* and *P*222 models.

**Figure 7:**
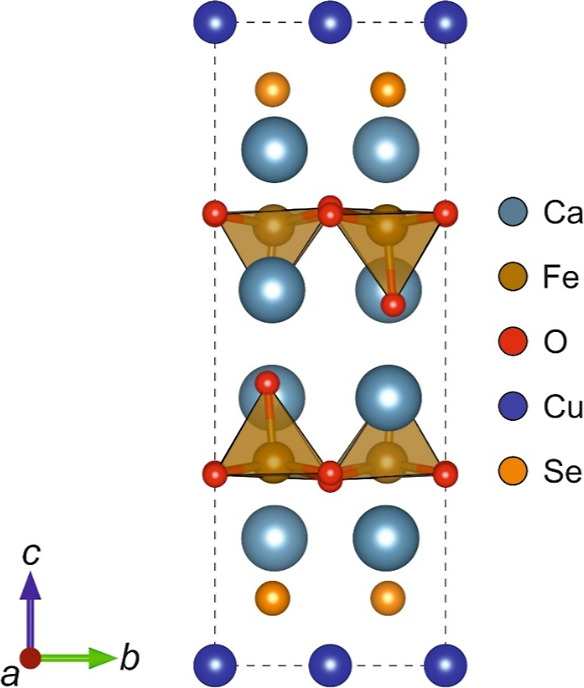
Proposed model in *P*4̅2*m* for the low-temperature distortion occurring in Ca_2_FeO_3_CuSe.

**Figure 8:**
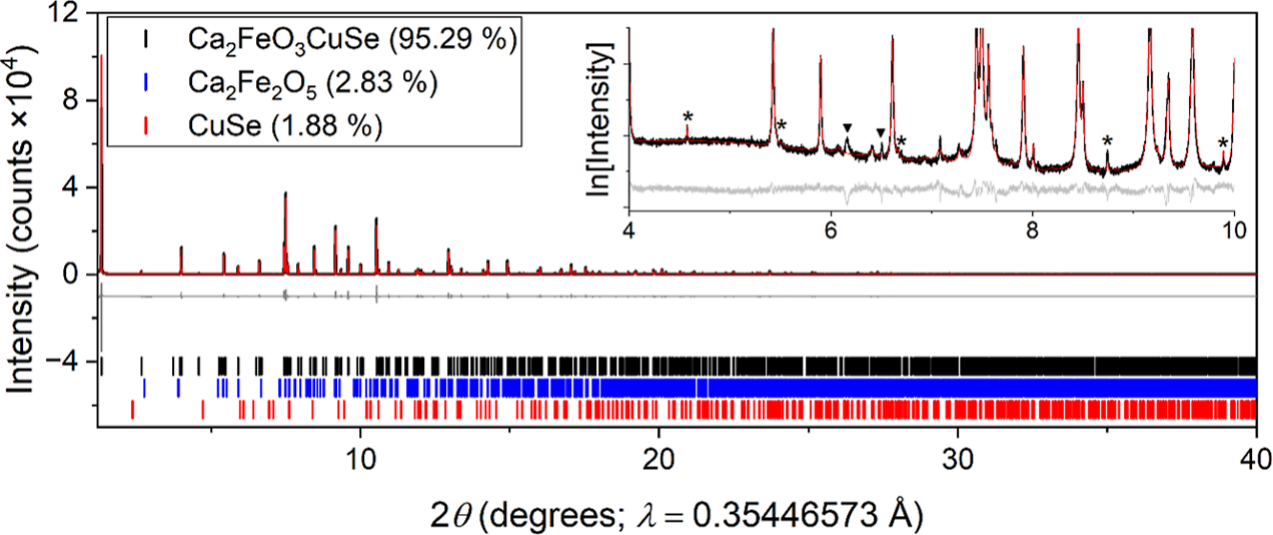
Rietveld refinement of Ca_2_FeO_3_CuSe using the *P*4̅2*m* structure model from data collected on the ID22 beamline at the ESRF.^20^ The peaks marked with an asterisk are those assigned to the superstructure and vary with temperature. The peaks marked with a black triangle do not vary in intensity with temperature and are assigned to a minuscule amount of unidentified impurity. The identified impurities seen in the ID22 data are not visible in the data collected on the I11 beamline (see Figure 1), where the data have a lower signal-to-noise ratio.

### Structure Refinement

Table 2 summarizes the ambient temperature structures of the four compounds obtained from the data in Figure 1. A comparison between data obtained from XRPD and NPD can be seen in Tables S1–S4.

**Table 2:** Refinement Results from XRPD Patterns Collected at 300 K Using the MAC Detector at I11

	Ca_2_FeO_3_CuSe	Ca_2_FeO_3_CuS	Sr_2_FeO_3_CuSe	Sr_2_FeO_3_CuS
space group	*P*4/*nmm*	*P*4/*nmm*	*P*4/*nmm*	*P*4/*nmm*
*R*_wp_ (%)	8.040	8.407	7.007	5.660
χ^2^	4.166	4.202	4.488	3.267
*a* (Å)	3.864130 (5)	3.82950 (1)	3.937591 (6)	3.901953 (5)
*c* (Å)	15.31524 (3)	14.95914 (7)	15.97075 (3)	15.643879 (3)
*V* (Å^3^)	228.679 (1)	219.377 (2)	247.620 (1)	238.182 (1)
Fe-*Ch* (Å) [1]^a^	3.2357 (8)	3.1650 (2)	3.4609 (8)	3.408 (1)
Cu-*Ch* (Å) [4]^a^	2.5085 (3)	2.4152 (9)	2.5105 (3)	2.4219 (6)
Fe–O(1) (Å) [4]^a^	1.9596 (4)	1.9434 (4)	1.9956 (4)	1.9779 (2)
Fe–O(2) (Å) [1]^a^	1.843 (3)	1.888 (4)	1.873 (4)	1.893 (2)
*Ch*-Cu-*Ch* α (deg) [2]^a^	100.74 (2)	104.89 (6)	103.30 (2)	107.12 (5)
*Ch*-Cu-*Ch* β (deg) [4]^a^	114.004 (9)	111.81 (3)	112.645 (10)	110.66 (3)
O(1)–Fe–O(2) γ (deg) [4]^a^	99.61 (6)	99.84 (7)	99.40 (7)	99.26 (5)
Fe BV sum^b^	3.123 (9)	3.14 (1)	2.85 (1)	2.910 (5)

a Numbers in square brackets indicate the number of bonds or angles of each type. The specific Fe–O bonds and *Ch*-Cu-*Ch* bond angles are defined in Figure 2b.

b Bond valence calculations were performed using literature data from Brown and Altermatt.^26^

The unit cell volumes and the *a* and *c* lattice parameters all behave as expected, increasing in value when ions of smaller ionic radii are replaced by those with larger ionic radii, such as going from Ca^2+^ to Sr^2+^ or from S^2–^ to Se^2–^. Another consequence of replacing Ca^2+^ with Sr^2+^ is the substantial increase in both Fe–O bond lengths, resulting in a decrease in the calculated Fe bond valence sum from +3.12 to +2.85 in the selenides and from +3.14 to +2.91 in the sulfides. The inclusion of the larger Sr^2+^ cation—with subsequent increase in the size of the unit cell in both the *a* and *c* directions—results in the elongation of both the Fe–O(1) bonds, within the basal plane, and the axial Fe–O(2) bond of the FeO_5_ square-based pyramids.

### Structural Distortions

Variable temperature XRPD scans were performed on all four compounds on the I11 beamline, and the behaviors of the lattice parameters are shown in Figures 3, S1, and S2. Analysis of the refined lattice parameters for Ca_2_FeO_3_CuSe and Ca_2_FeO_3_CuS reveals subtle distortions occurring in the crystal structures, which are not observed for the strontium analogues, which displayed a smooth trend in their structural parameters on cooling, consistent solely with thermal contraction. At temperatures slightly lower than 300 K for Ca_2_FeO_3_CuSe and 200 K for Ca_2_FeO_3_CuS, transitions occur, which result in the *c* lattice parameter decreasing in value significantly faster than the *a* lattice parameter as the temperature of the sample decreases, while the unit cell volume contracts smoothly (Figure S2). This is clearly illustrated by the large change in the *c*/*a* ratios (see Figure 3) and initiated an investigation into the nature of these distortions.

ED measurements performed on Ca_2_FeO_3_CuSe show that, upon cooling from RT to 100 K, superstructure reflections are present, developing from weak diffuse streaks present at (*h*/2 *k*/2 *l*) at RT, as shown in Figure 4. These extra reflections can be indexed on a √2*a* × √2*a* × *c* expansion of the parent cell. 3D ED data acquired at 100 K on a 200 nm crystallite of Ca_2_FeO_3_CuSe allowed reconstruction of the [001], [100], and [110] zones; these matched with the direct in-zone SAED patterns and revealed further weak reflections consistent with the expanded cell, and no reflection conditions were evident. Further details and ED results can be seen in Figures S3–S8.

Figure 5 shows how very weak supercell peaks also emerge in the variable temperature XRPD data as the sample is cooled, which can be indexed on the same √2*a* × √2*a* × *c* expansion of the parent cell. ISODISTORT^27^ was utilized to analyze the various positional displacements that are available to the structure using this supercell.

This produced four candidate space groups in the expanded cell: *P*4̅2m (no. 111), *P*4*mm* (no. 99) for tetragonal symmetry and *P*222 (no. 16) and *Pmm*2 (no. 25) for orthorhombic symmetry. The models with space group *Pmm*2 (no. 25) and *P*4*mm* (no. 99) are derived from the parent cell by the oxygen breathing mode M3, which permits the simultaneous expansion and contraction of some Fe–O bonds, which would result in crystallographically distinct Fe sites in a checkerboard arrangement and would be suggestive of charge ordering, which is not consistent with the single Fe-oxidation state in these compositions. In contrast, the models with space group *P*222 (no. 16) and *P*4̅2*m* (no. 111) are derived from the parent cell by the oxygen tilting mode M2, which describes the buckling of the apical Fe–O(1) with an accompanying displacement of the basal Fe–O(2) bonds, as depicted in Figure 6. This polyhedral tilting is similar to that commonly found in perovskite oxides.

Structure solution from the powder data for Ca_2_FeO_3_CuSe obtained from the data collected on ID22 was performed using the charge flipping algorithm in SUPERFLIP, as implemented in Jana 2020,^24^ and was consistent with the two candidate space groups *P*222 (no. 16) and *P*4̅2*m* (no. 111). Rietveld refinements were performed against powder data collected at 81 K, with both models featuring similar tilting distortions of the oxide polyhedra and gave fits of comparable statistical quality (for *P*222, *R*_wp_ = 2.953% with 13 atoms, and for *P*4̅2*m*, *R*_wp_ = 2.986% with 11 atoms). The apparent retention of the 4-fold axis from the ED images and a lack of peak splitting in the high-resolution powder data from ID22 lead to the conclusion that the model in space group *P*4̅2*m* is more appropriate. The model and Rietveld refinement against the experimental data are shown in Figures 7 and 8.

There are multiple factors that explain why this is the most probable model. There are no signs of nonstoichiometry when the occupancy of each site is refined, as expected in the absence of significant impurity phases (see Figure 1), so this rules out the possibility that the superstructure arises from vacancy ordering. The fact that these superstructure reflections are very weak (so much so that they can only be identified using ED and high-intensity synchrotron radiation and when the data are plotted on a logarithmic scale (Figure 5) is consistent with them being due to the movement of the O^2–^ ions.

ED measurements were also performed on a sample of Ca_2_FeO_3_CuS and, similar to the Ca_2_FeO_3_CuSe case, show that upon going from ambient temperature to 100 K, superstructure reflections appear in the diffraction patterns in addition to reflections produced by the undistorted phase, as depicted in Figure 9. These extra reflections can also be indexed on a √2*a* × √2*a* × *c* expansion of the parent cell, although the *hk*0: *h* + *k* = 2*n* reflection condition was suggested from the patterns, giving the extinction symbol *Pn*--. However, the *n*-glide plane perpendicular to the stacking direction of the layers is inconsistent with the arrangement of the double layers of basal-vertex-linked FeO_5_ pyramids in the expanded cell (see Figure S9). The strongest superstructure reflections (assuming the low-temperature structure to be similar to the Ca_2_FeO_3_CuSe case) are much weaker in Ca_2_FeO_3_CuS than in Ca_2_FeO_3_CuSe, and the *hk*0: *h* + *k* ≠ 2*n* reflections are already barely visible in Ca_2_FeO_3_CuSe, and so it is probable that the *hk*0: *h* + *k* ≠ 2*n* reflections in Ca_2_FeO_3_CuS are too weak to be observed in the diffraction data.

**Figure 9:**
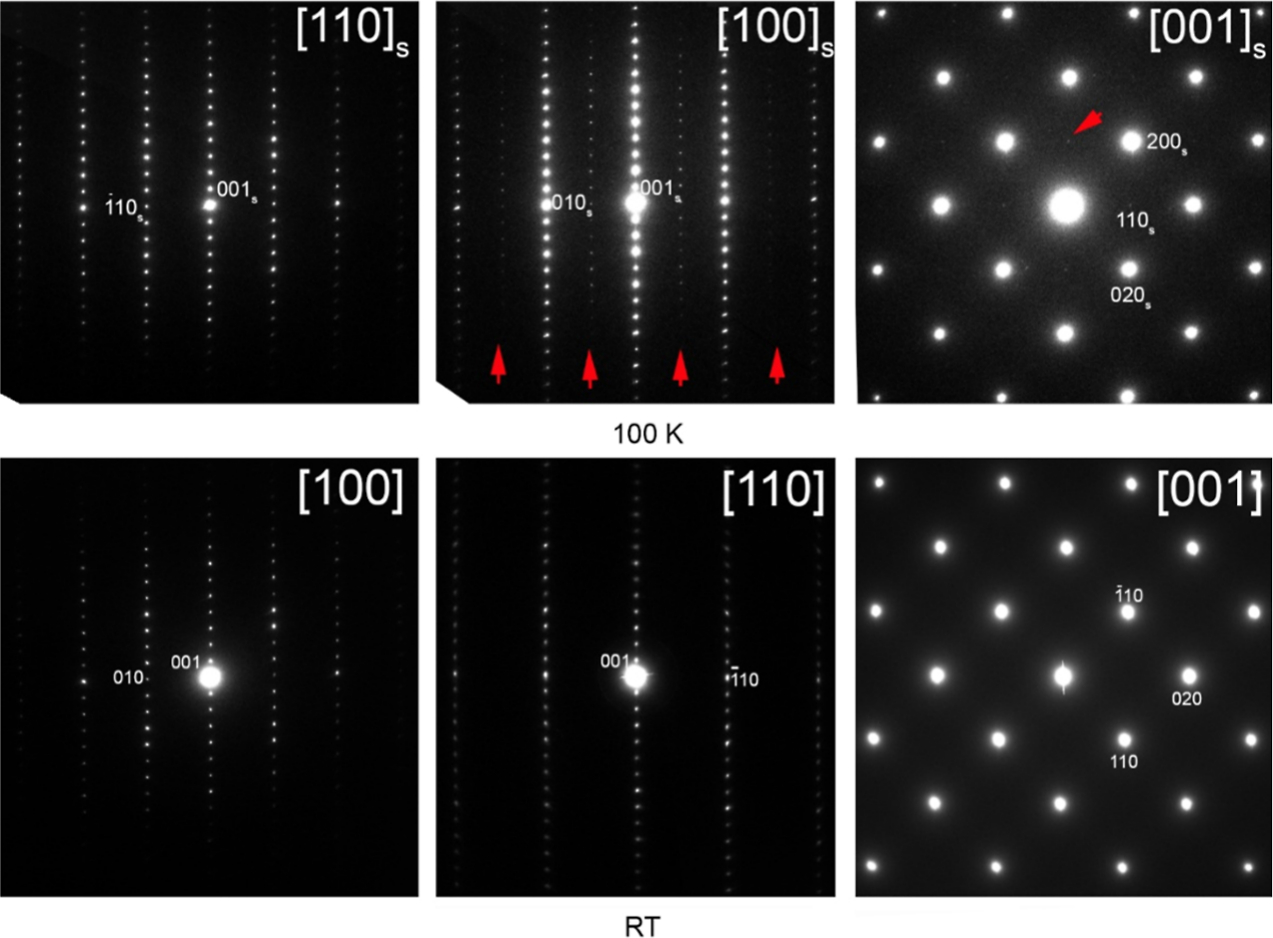
Comparison of various SAED zones observed for Ca_2_FeO_3_CuS upon cooling from 300 to 100 K. The subscript s denotes zones and diffraction spots belonging to the √2*a* × √2*a* × *c* supercell. The red arrows highlight the appearance of new diffraction spots at 100 K. Note that the reflection labeled as 010 in the ambient temperature [100] zone appears due to double diffraction.

Due to the very similar nature of the small superstructure peaks observed in XRPD (Figure 10) and the very similar rate at which the *c*/*a* ratio changes (approximate change of 0.01 per 100 K) when compared to Ca_2_FeO_3_CuSe, it seems realistic that the same distortion of the apical O^2–^ is occurring. Indeed, the *P*4̅2m model as used for the selenide also accounts for the superstructure peaks in the sulfide, with a Rietveld plot shown in Figure 11. We note that the distortion of the structure on cooling involves significant displacement of the oxide ions from their positions in the RT structures, and these atoms make a small contribution to the X-ray intensities. NPD would offer much greater sensitivity to the oxide positions, but in this case, because the magnetic ordering (see below) involves a similar cell expansion to the structural distortion and the structural distortion occurs below the magnetic ordering temperature, there is overlap of the new structural reflections and the magnetic reflections in the neutron diffraction patterns, at least at longer *d*-spacings, which hampers these neutron powder data from providing greater structural insight. The *P*4̅2*m* low-temperature structural model refined stably against the low-temperature X-ray and neutron data (Table S3a–e), unlike other attempted models.

**Figure 10:**
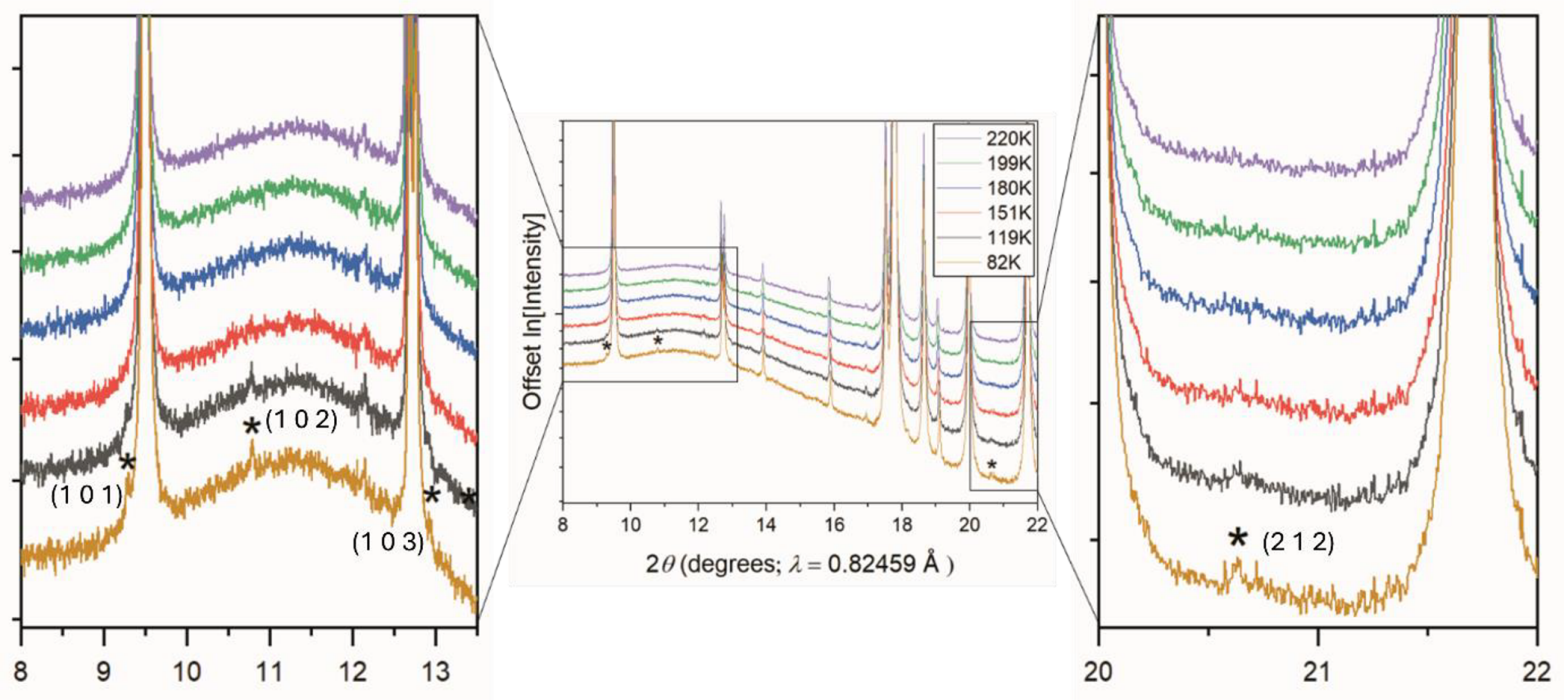
Evolution of weak supercell peaks (highlighted by a * and indices included) present in the variable temperature XRPD data from I11 (DLS) of Ca_2_FeO_3_CuS.

**Figure 11:**
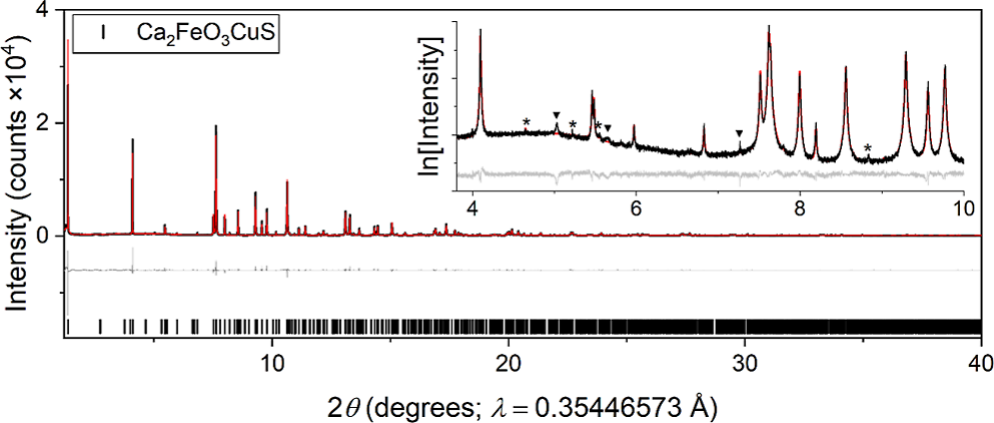
Rietveld refinement of Ca_2_FeO_3_CuS using the *P*4̅2*m* structure model against ID22 (ESRF) data at 4 K. The peaks marked with an asterisk in the inset (on a logarithmic scale) are those assigned to the superstructure and vary with temperature. The peaks marked with triangles do not vary with temperature and cannot be indexed and are assumed to be a minuscule amount of unidentified impurity.

Tables 3 and S3a–e summarize the structural refinements against low-temperature synchrotron X-ray and NPD data.

**Table 3:** Comparison of the Low *T* Structural Model Obtained Using X-ray and Neutron Radiation for Ca_2_FeO_3_CuSe and Ca_2_FeO_3_CuS

	Ca_2_FeO_3_CuSe		Ca_2_FeO_3_CuS	
radiation type	X-ray (ESRF)	neutron (WISH)	X-ray (ESRF)	neutron (WISH)
temperature (K)	4	8	4	8
space group	*P*4̅2*m*	*P*4̅2*m*	*P*4̅2*m*	*P*4̅2*m*
*R*_wp_ (%)	9.52	7.54	5.41	3.67
χ^2^	3.22	0.10	1.60	0.05
*a* (Å)	5.458390 (8)	5.46404 (3)	5.407963 (7)	5.41141 (8)
*c* (Å)	15.22527 (4)	15.2466 (1)	14.88044 (3)	14.8955 (3)
*V* (Å^3^)	453.612 (2)	455.199 (6)	435.194 (2)	436.19 (2)
Fe-*Ch* (Å) [1]	3.2142 (6)	3.196 (4)	3.1461 (7)	3.100 (5)
Fe–O(1) (Å) [2]	1.966 (9)	1.964 (2)	1.946 (2)	1.939 (2)
Fe–O(2) (Å) [1]	2.003 (2)	1.994 (7)	1.986 (2)	2.004 (6)
Fe–O(3) (Å) [1]	1.896 (3)	1.964 (5)	1.885 (2)	1.902 (6)
Fe–O(4) (Å) [1]	1.911 (2)	1.906 (5)	1.908 (2)	1.899 (4)

### Magnetic Ordering

Magnetometry data collected at and below RT (and hence below the transitions to long-range magnetic order) on each sample were uninformative regarding the magnetic ordering (see captions to Figures S10 and S11), and therefore, NPD was the key technique in elucidating the magnetic interactions in the *Ae*_2_FeO_3_Cu*Ch* (*Ae* = Ca, Sr; *Ch* = Se, S) family of compounds and is discussed in the following sections divided into the Ca-containing and Sr-containing compounds.

### Ca_2_FeO_3_CuSe and Ca_2_FeO_3_CuS

Extra intense Bragg reflections are present in the NPD data for Ca_2_FeO_3_CuSe and Ca_2_FeO_3_CuS collected at 8 K, highlighted by a * in Figures 12 and 13, which cannot be accounted for by scattering from only the nuclear model (we note here that we did not observe measurable nuclear or magnetic reflections in the data on Ca_2_FeO_3_CuSe attributable to the small brownmillerite Ca_2_Fe_2_O_5_ impurity noted in Figure 8). These large *d*-spacing reflections can be indexed on a unit cell that is a √2*a* × √2*a* × *c* expansion of the nuclear unit cell (i.e., similar to the low-temperature structural cell expansion) and arise from long-range magnetic ordering of the Fe^3+^ moments. Analysis of the NPD data was carried out using the ISODISTORT software to generate possible magnetic irreducible representations with subsequent Rietveld refinement of the possible modes against the data. The ordering of the Fe^3+^ moments in both of these Ca-containing compounds can be best modeled by a combination of the mΓ_3_(a) and mΓ_5_(a) antiferromagnetic modes—as depicted in Figure 14—where the moments are tilted away from the crystallographic axes (see Figure S12 for a visual representation of all individual modes).

**Figure 12:**
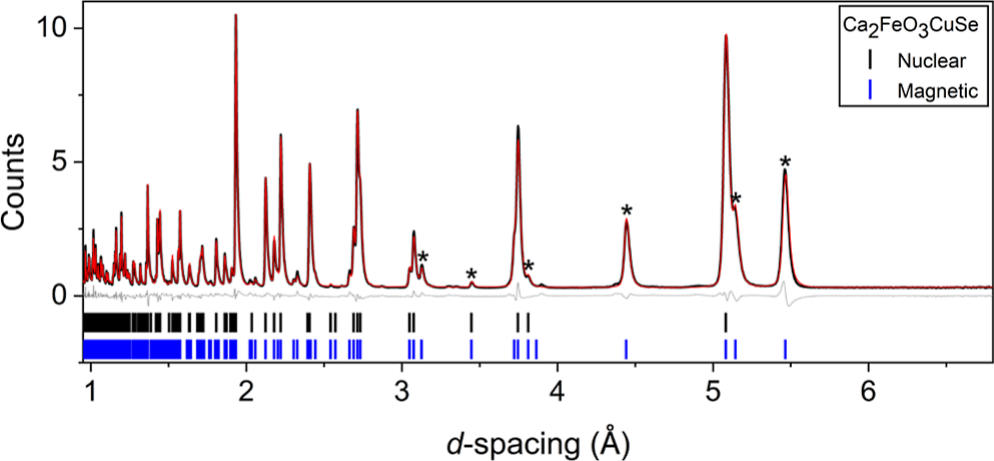
NPD pattern of Ca_2_FeO_3_CuSe (combination of banks 3 and 8 with average 2θ = 90°) measured at 8 K on the WISH instrument at ISIS showing the observed (black), calculated (red), and difference (gray) curves. Each * denotes a magnetic Bragg peak. *R*_wp_: 5.595%.

**Figure 13:**
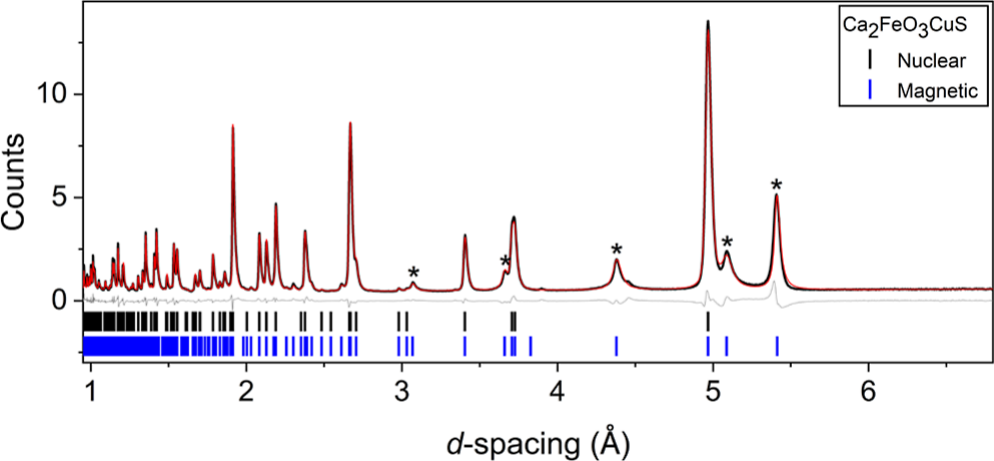
NPD pattern of Ca_2_FeO_3_CuS (combination of banks 3 and 8 with average 2θ = 90°) measured at 8 K on the WISH instrument at ISIS showing the observed (black), calculated (red), and difference (gray) curves. Each * denotes a magnetic Bragg peak. *R*_wp_: 5.661%.

**Figure 14:**
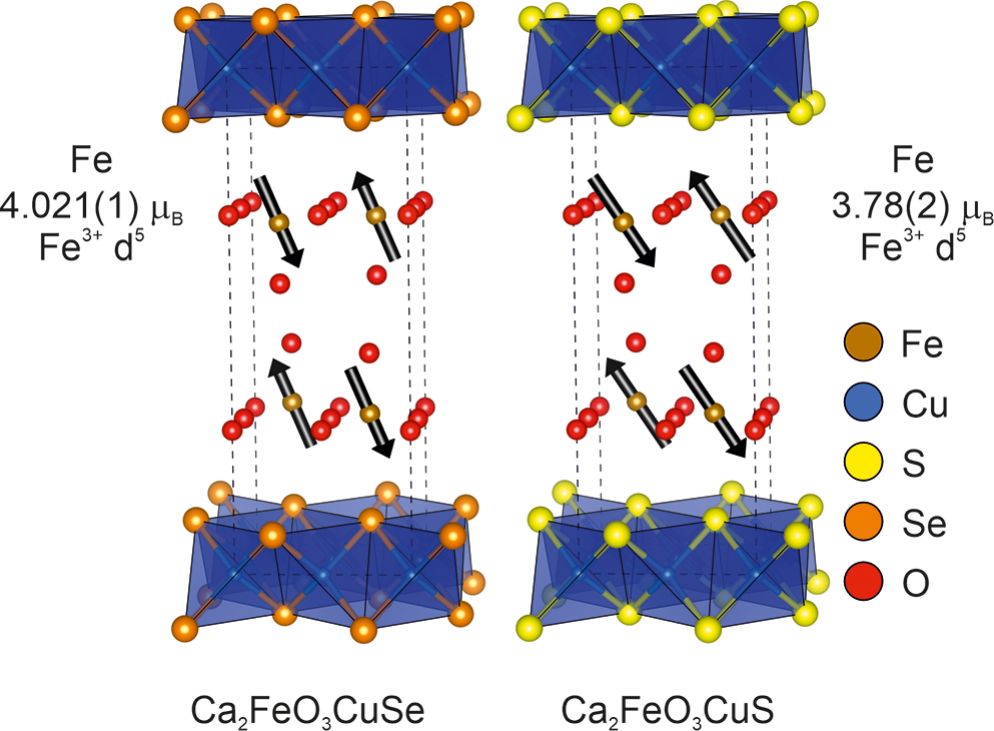
Models for the magnetic order in Ca_2_FeO_3_CuSe and Ca_2_FeO_3_CuS at 8 K, refined using data collected on the WISH instrument at ISIS. Ca ions are omitted for clarity. The magnetic supercell is indicated by the dashed lines. For this magnetic structure, a powder diffraction measurement is only able to determine the orientation of the moment relative to the *c*-axis.^28^

Figure S13 shows a plot of how the angle of the Fe^3+^ moment relative to the *c*-axis evolves with temperature for both the selenide and sulfide. Upon warming from 8 K, a slight spin reorientation can be seen toward the *c*-axis for the selenide and toward the *ab*-plane for the sulfide. The magnetic moment per Fe^3+^ ion tends toward similar values of 4 μ_B_ and 3.75 μ_B_ for the selenide and sulfide, respectively, on cooling (see Figure 15). A degree of covalent character in the Fe–O bonds is responsible for the reduction from 5 μ_B_ that would be expected for a high spin *d* ^5^ cation and the difference in the saturated magnetic moments is consistent with the bond valence sums of the Fe^3+^ cations with the iron ions being slightly more under-bonded in Ca_2_FeO_3_CuSe compared with the sulfide analogue (see Table 2) and hence having less covalency and a larger magnitude of the moment localized on the Fe^3+^ center.

**Figure 15:**
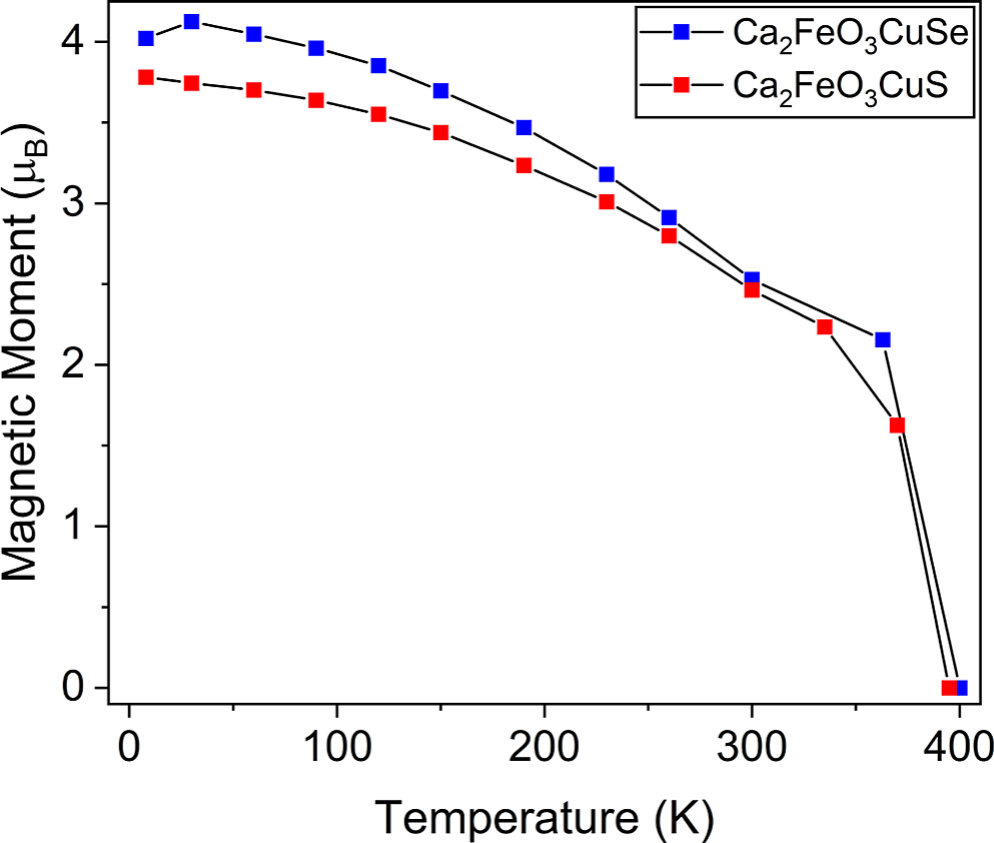
Total magnetic moment of Ca_2_FeO_3_CuSe and Ca_2_FeO_3_CuS at different temperatures, refined using data collected on the WISH instrument at ISIS.

A Néel temperature (*T*_N_) of 390(10) K can be estimated for both compounds from the disappearance of the magnetic Bragg peaks by 400 K. This similarity in the strength of the interactions between the magnetic ions in both materials is again due to the comparable lattice parameters and Fe–O bond distances.

### Sr_2_FeO_3_CuSe and Sr_2_FeO_3_CuS

In the case of Sr_2_FeO_3_CuSe, low-temperature NPD data collected at 1.5 K reveal magnetic Bragg peaks, which can be indexed on a unit cell that is a √2*a* × √2*a* × 2*c* expansion of the nuclear unit cell. The magnetic unit cell is doubled along the stacking direction compared with the Ca-containing cases.

The peaks for this low-temperature magnetic structure can be accounted for by the activation of the mM_1_(a) and mA_4_(a) magnetic modes, and the refinement is shown in Figure 16. Figure 17 depicts the refined magnetic model for this material. In this case, the arrangement of the moments within each oxide slab is similar to those found for the Ca analogues, described above, but the arrangement of alternate slabs is different, and hence, the magnetic cell has a doubling of the *c*-axis compared with the nuclear cell. The saturated total magnetic moment at 1.5 K is 4.07(8) μ_B_ per Fe^3+^ ion in Sr_2_FeO_3_CuSe, very similar to that in the Ca analogue.

**Figure 16:**
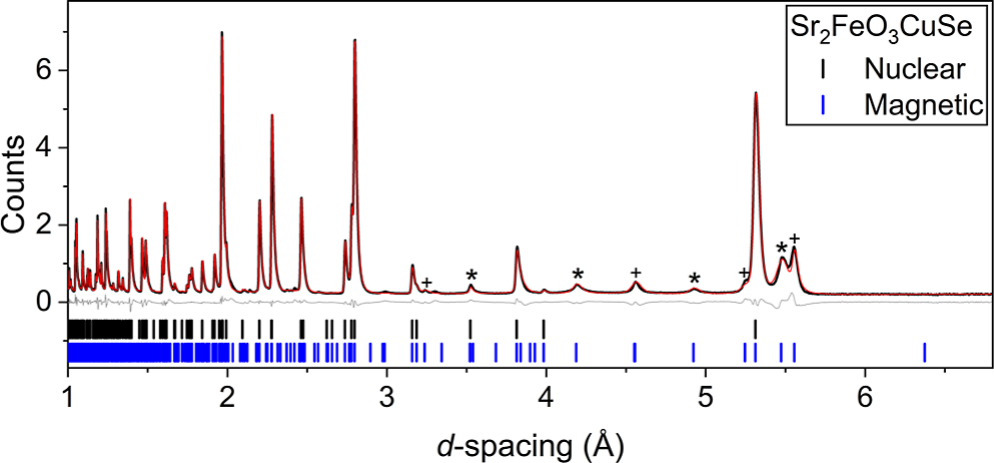
NPD pattern of Sr_2_FeO_3_CuSe (combination of banks 3 and 8 with average 2θ = 90°) measured at 1.5 K on the WISH instrument at ISIS showing the observed (black), calculated (red), and difference (gray) curves. *R*_wp_: 4.48%. The peaks marked with an * are from the mA_4_(a) magnetic mode contribution, and the peaks marked with a + are from the mM_1_(a) magnetic modes contribution. The + peaks remain above RT. See Figure S12 for a visual representation of these individual magnetic modes.

**Figure 17:**
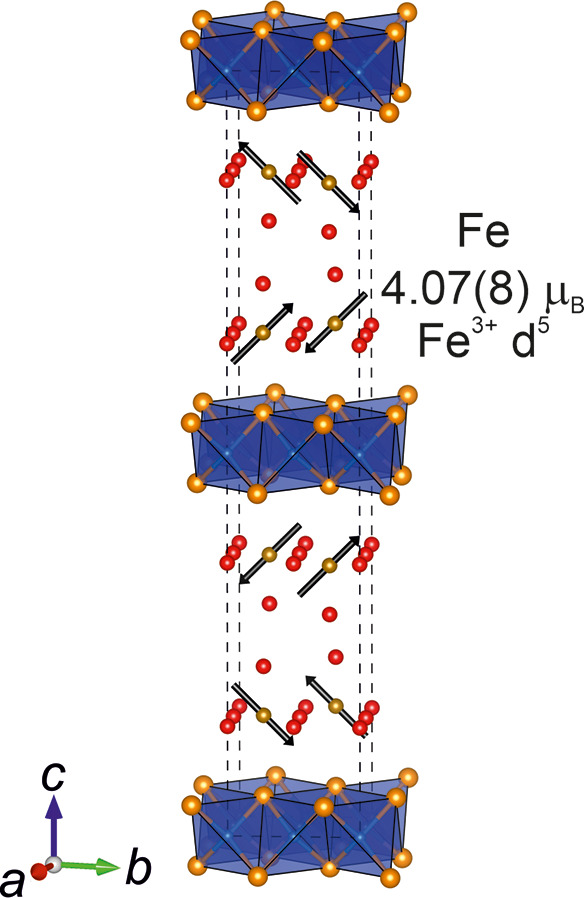
Model for the magnetic order in Sr_2_FeO_3_CuSe, refined using data collected at 1.5 K on the WISH instrument at ISIS. The magnetic supercell is indicated by the dashed lines. For this magnetic structure, a powder diffraction measurement is only able to determine the orientation of the moment relative to the *c*-axis.^28^

On warming Sr_2_FeO_3_CuSe from 1.5 to 150 K, the Bragg peaks that carry intensity as a result of activation of the mA_4_(a) mode [marked with (*)] decrease in intensity much faster than those that carry intensity due to activation of the mM_1_(a) mode [marked with (+)]. This is most noticeable in the high *d*-spacing region at around *d* = 5.5 Å, as shown in Figure 18. The drop in intensity of the mA_4_(a) peaks occurs over this whole temperature range, but it is most noticeable from 100 to 150 K. As the mM_1_(a) mode corresponds to moment components directed within the *ab*-plane and the mA_4_(a) to moment components directed along the *c*-axis, this disappearance of the mA_4_(a) contribution suggests that a spin-reorientation of the tilted moments is occurring toward the *ab*-plane (see Figure S12 for a visual representation of individual magnetic modes).

**Figure 18:**
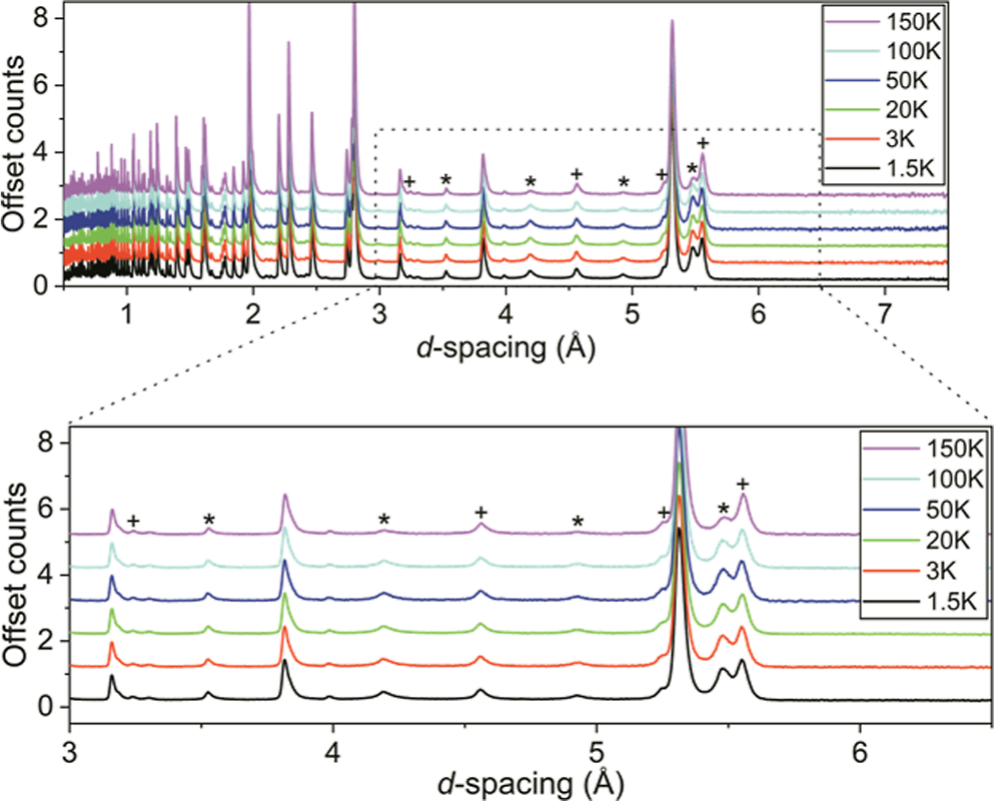
NPD patterns of Sr_2_FeO_3_CuSe (combination of banks 3 and 8 with average 2θ = 90°) measured at 1.5 K, 3 K, 20 K, 50 K, 100 K, and 150 K on the WISH instrument at ISIS. Each * denotes a magnetic Bragg peak modeled by mA_4_(a) and each + denotes a magnetic Bragg peak modeled by mM_1_(a).

High-temperature NPD studies were later carried out on D2B at the ILL^22^ to explore this moment-reorientation further (see Figures S14 and S15 for additional data). On warming to 230 K, the peaks denoted by (*) corresponding to the mA_4_(a) mode are no longer evident; hence, the spin-reorientation is complete, and the magnetic model in the region immediately below the transition is comprised of the mM_1_(a) mode only with the Fe^3+^ moments directed in the *ab*-plane. Figure 19 depicts that the component of the Fe^3+^ moment along the *c*-axis ceases to contribute at some temperature between 150 and 230 K and also shows how the remaining magnetic Bragg peaks decrease in intensity from 230 K to 298 K and are no longer present by 400 K, leading to an estimated *T*_N_ of 370(30) K. Figure 20 summarizes the key changes in the magnetic model of Sr_2_FeO_3_CuSe from 1.5 to 230 K; as the spin reorientation is complete, the doubling of the magnetic cell along *c* is no longer required. The magnetic evolution from 1.5 to 300 K is included for comparison and is subsequently discussed in the next section.

**Figure 19:**
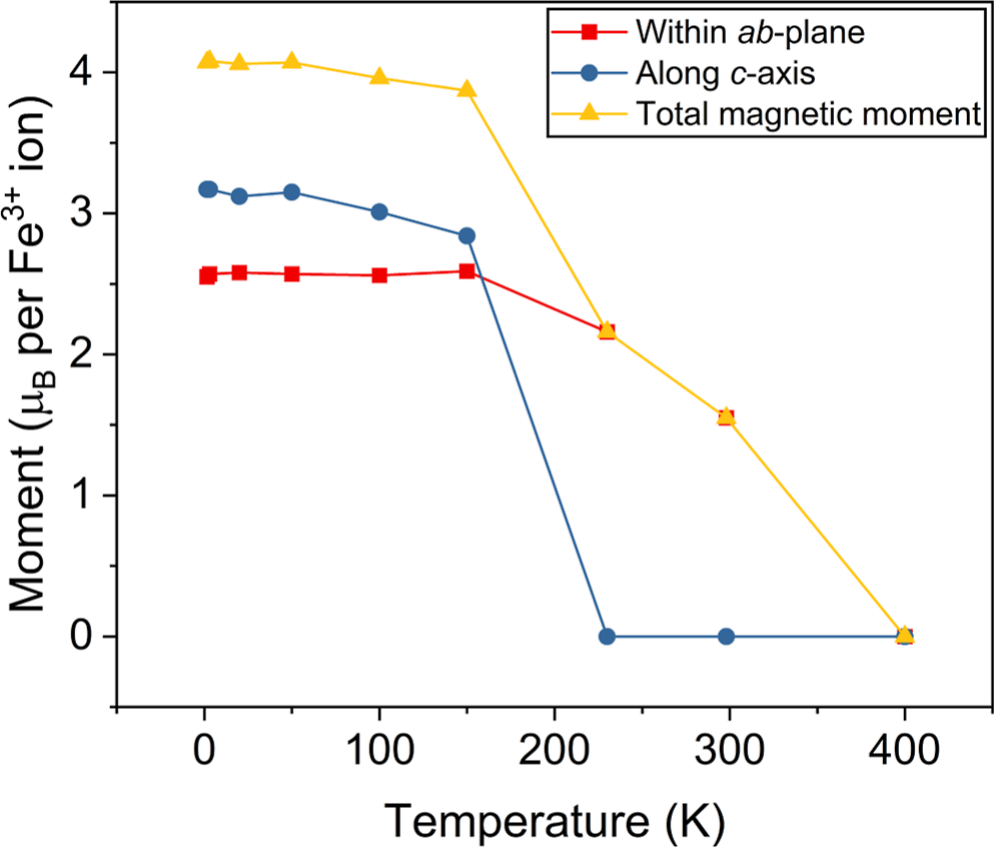
Total magnetic moment of Sr_2_FeO_3_CuSe at different temperatures, along with the contributions to the total moment within the *ab*-plane and along the *c*-axis, refined using data collected on the WISH instrument at ISIS (1.5–150 K) and the D2B instrument at the ILL (230–400 K).

**Figure 20:**
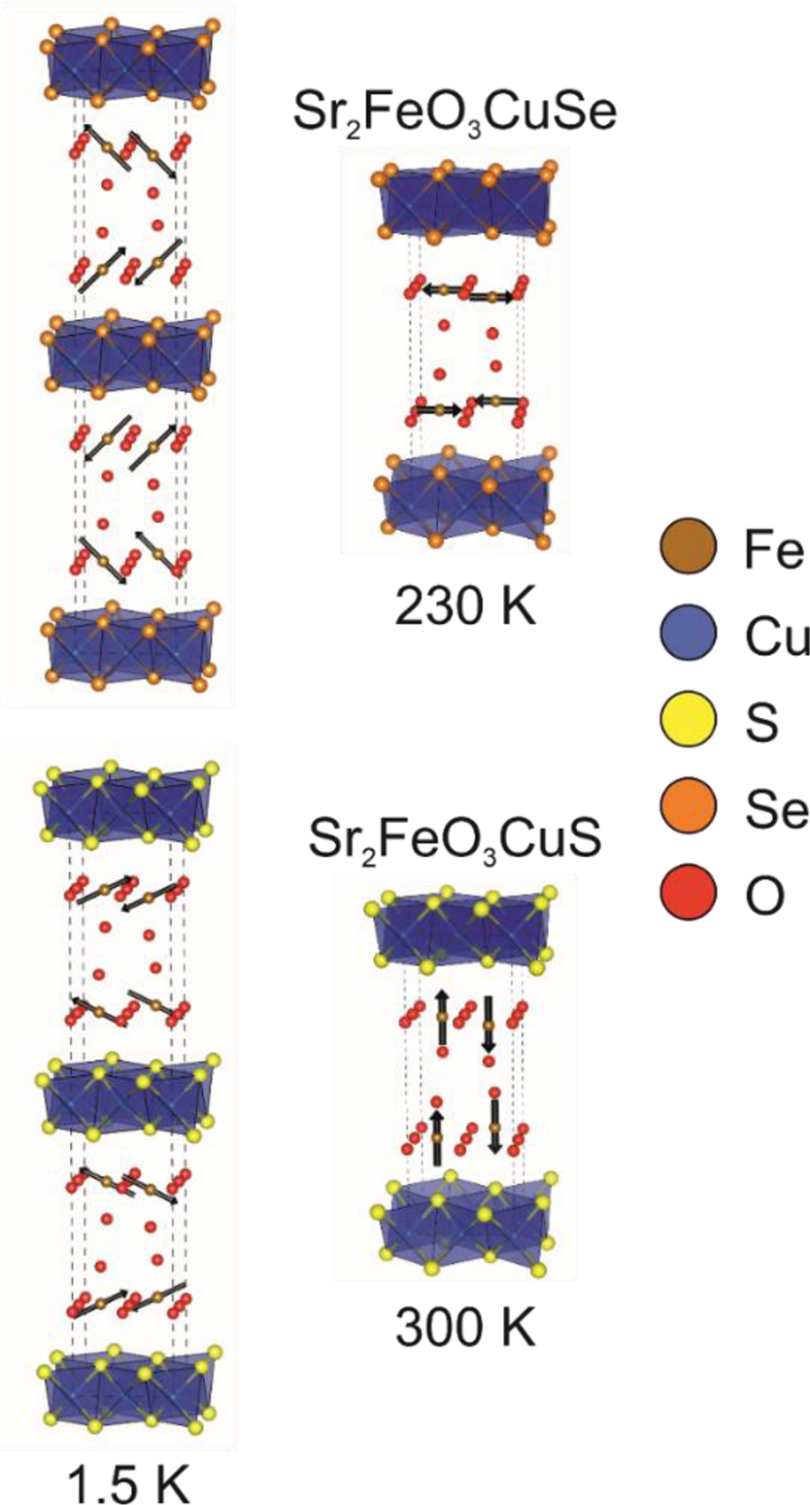
Models for the magnetic order in Sr_2_FeO_3_CuSe and Sr_2_FeO_3_CuS at different temperatures, refined using data collected on the WISH instrument at ISIS (1.5 K for the selenide and both temperatures for the sulfide) and the D2B instrument at the ILL (230 K). The spin reorientation is already complete at the higher temperature, and thus, the magnetic cell is no longer doubled. The magnetic cells are indicated by the dashed lines. For these magnetic structures, a powder diffraction measurement is only able to determine the orientation of the moment relative to the *c*-axis.^28^

For the oxysulfide analogue Sr_2_FeO_3_CuS, at RT, magnetic Bragg peaks in the NPD data can be indexed using a √2*a* × √2*a* × *c* expansion of the nuclear unit cell. These data collected just below the transition can again be modeled by a single magnetic mode—in this case the mM_4_(a) mode, which directs the moments on the Fe centers parallel to the *c*-axis. So, this is in direct contrast with Sr_2_FeO_3_CuSe, in which the moments are perpendicular to the *c*-axis (Figure 20). Upon cooling to 20 and 1.5 K, a unit cell that is a √2*a* × √2*a* × 2*c* expansion of the nuclear unit cell is necessary to account for all reflections of Sr_2_FeO_3_CuS (see Figure S16 for further data), and at these lower temperatures, the mA_2_(a) mode is also activated in addition to mM_4_(a), so the Fe^3+^ moments are now tilted away from the *c*-axis and toward the *ab*-plane. Therefore, there is evidence that a significant spin reorientation as a function of temperature occurs here and that the reorientation proceeds in the opposite direction to the one present in Sr_2_FeO_3_CuSe as a function of temperature. The refined long-range ordered magnetic moment per Fe^3+^ in the Sr_2_FeO_3_CuS compound is 3.42(1) μ_B_ at 1.5 K, with Rietveld refinement of the 1.5 K data shown in Figure 21. Sr_2_FeO_3_CuSe and Sr_2_FeO_3_CuS thus have qualitatively similar low-temperature magnetic structures, but with different orientations of the moments relative to the *c*-axis.

**Figure 21:**
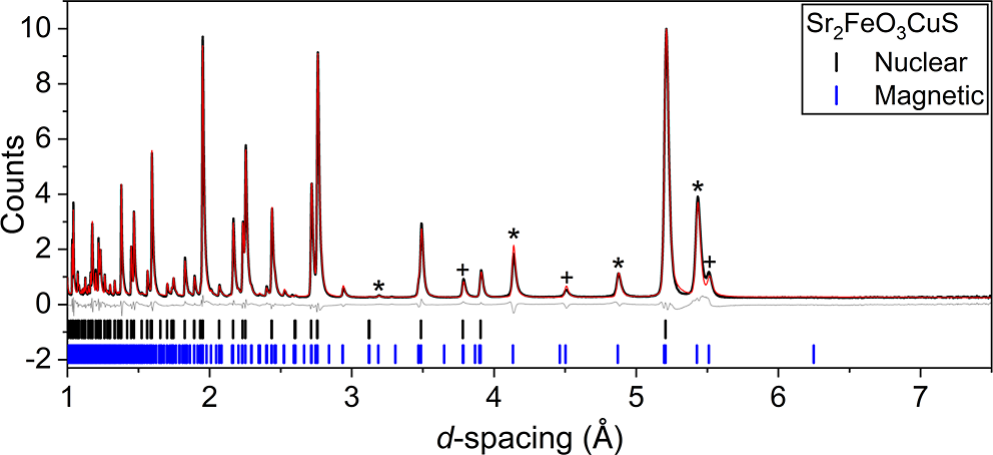
NPD pattern of Sr_2_FeO_3_CuS (combination of banks 3 and 8 with average 2θ = 90°) measured at 1.5 K on the WISH instrument at ISIS showing the observed (black), calculated (red), and difference (gray) curves. *R*_wp_: 5.624%.

### Magnetic Ordering Comparisons

Here, we summarize the similarities and differences in the magnetic models of the four compounds deduced from the magnetic reflections and their intensities (see Tables 2 and 4). In all four compounds, the magnetic Fe^3+^ ions are in oxide slabs made of double layers of FeO_5_ square pyramids, and each pyramid shares its four basal vertices with neighboring similar polyhedra, providing approximately 180 °Fe–O–Fe superexchange pathways (refer to Figure 2a). Two magnetic modes are required in combination at low temperature for all four materials, and the moments lie at some angle between the *ab* plane and the *c*-axis in an arrangement dominated by the strong ∼180 °Fe–O–Fe antiferromagnetic superexchange (consistent with the Goodenough–Kanamori rules^29–31^ ). Ca_2_FeO_3_CuSe and Ca_2_FeO_3_CuS both have an AAAA-type stacking of the arrangement of the ordered magnetic moments within these slabs (Figure 14), and hence, there is no doubling of the *c*-axis of the magnetic unit cell relative to the nuclear cell in the high- and low-temperature magnetic structures. In the high-temperature (structurally undistorted) regime, they were found to have the *P*_*A*_2_1_/*c* magnetic space group (14.83) in the Belov, Neronova, and Smirnova (BNS) scheme [*C*_*P*_2′/*m*′ (12.11.76) in the Opechowski and Guccione (OG) scheme].^32^ In the low-temperature regime, where the structures are subtly distorted, they were found to have the P2′ magnetic space group (3.3) in the BNS scheme [P2′ (3.3.10) in the OG scheme].^32^ In contrast, in the low-temperature magnetic structures of Sr_2_FeO_3_CuSe and Sr_2_FeO_3_CuS, the slabs are stacked in an ABAB fashion, and there is doubling of the periodicity in the *c*-direction relative to the nuclear cell (Figure 20). In all four cases, there is some reorientation of the moments as a function of temperature (Table 4), and we treat these spin reorientations as continuous. Higher temperature resolution in the neutron diffraction measurements would be required to confirm definitively whether these spin reorientations are continuous or abrupt. In the cases of Ca_2_FeO_3_CuSe and Ca_2_FeO_3_CuS, the reorientation angles are small, while in Sr_2_FeO_3_CuSe and Sr_2_FeO_3_CuS, the reorientations are much larger and in opposing directions, as shown in Figure 20 above. Furthermore, in Sr_2_FeO_3_CuSe and Sr_2_FeO_3_CuS, only a single M-point magnetic mode is required at high temperatures, just below the long-range magnetic ordering transition, and here, there is no doubling of the *c* lattice vector in the description of the magnetic structure (so at these higher temperatures, the stacking of the magnetically ordered slabs is AAAA), resulting in the *P*_A_ *bcm* magnetic space group (57.389) in the BNS scheme [*C*_P_ *mma*′ (67.15.591) in the OG scheme] for Sr_2_FeO_3_CuSe and the *P*_B_ *cca* magnetic space group (54.350) in the BNS scheme [*C*_P_ *m*′*ma* (67.13.589) in the OG scheme] for Sr_2_FeO_3_CuS in this higher temperature part of the magnetically ordered regime. In both Sr-containing cases, activation of an A-point magnetic mode on further cooling (below RT) results in a doubling of the *c* lattice vector in the description of the long-range magnetic ordering and the ABAB-type stacking of the magnetically ordered slabs at the lowest temperatures, resulting in models in the *P*_C_ *bca* magnetic space group (61.439) in the BNS scheme [*C*_P_ *m*′*ca*′ (64.16.543) in the OG scheme] for Sr_2_FeO_3_CuSe and the *P*_A_ *bcn* magnetic space group (60.429) in the BNS scheme [*C*_P_ *m*′*c*′*a*′ (64.17.544) in the OG scheme] for Sr_2_FeO_3_CuS.

**Table 4:** Comparison of the Nuclear Unit Cell Expansions Necessary for the Magnetic Models, the Magnetic Modes Present at Different Temperatures along with the Direction and Magnitude of the Spin-Reorientations and the Magnetic Moments in the *Ae*_2_FeO_3_Cu*Ch* (*Ae* = Ca, Sr; *Ch* = S, Se) Compounds, along with Their Associated Néel Temperatures

	Ca_2_FeO_3_CuSe	Ca_2_FeO_3_CuS	Sr_2_FeO_3_CuSe	Sr_2_FeO_3_CuS
low *T* expansion of the RT structural cell	√2*a* × √2*a* × *c*	√2*a* × √2*a* × *c*	√2*a* × √2*a* × 2*c*	√2*a* × √2*a* × 2*c*
activated modes	mΓ_3_(a)/mΓ_5_(a)	mΓ_3_(a)/mΓ_5_(a)	mM_1_(a)/mA_4_(a)	mM_4_(a)/mA_2_(a)
BNS low *T* space group	*P*2′	*P*2′	*P*_C_ *bca*	*P*_A_ *bcn*
RT expansion of the RT structural cell	√2*a* × √2*a* × *c*	√2*a* × √2*a* × *c*	√2*a* × √2*a* × *c*	√2*a* × √2*a* × *c*
activated modes	mM_1_(a)/mM_4_(a)	mM_1_(a)/mM_4_(a)	mM_1_(a)	mM_4_(a)
BNS RT space group	*P*_A_2_1_/*c*	*P*_A_2_1_/*c*	*P*_A_ *bcm*	*P*_B_ *cca*
spin reorientation	*c*-axis	*ab*-plane	*ab*-plane	*c*-axis
angle change	9.3 (9) °	5.8 (10) °	39.2°	64.3°
saturated Fe moment (μ_B_)	4.02 (1) at 8 K	3.78 (2) at 8 K	4.07 (8) at 1.5 K	3.42 (1) at 1.5 K
^a^estimated *T*_N_ (K)	390 (10)	390 (10)	370 (30)	insufficient data

a Estimated from the temperature evolution of magnetic Bragg scattering.

The orientation of localized transition-metal magnetic moments relative to the ligand field can often be predicted using spin–orbit coupling arguments, as described by Whangbo et al.^33^ However, in the case of Fe^3+^ (high-spin d^5^), there is no significant orbital contribution and hence no strong orientational preference, as has been found in other Fe^3+^ compounds.^13^

Table 4 compares the saturated moments determined at the lowest temperatures used and the estimated *T*_N_ values for these materials (from the temperature evolution of the magnetic scattering). The selenides have larger saturated magnetic moments than the sulfides, due to the lower iron bond valence sum, which results in a higher degree of electron density localized on the Fe^3+^ ions because there is less covalency in the longer Fe–O bonds, which result from the incorporation of the larger chalcogenide. The comparable values for *T*_N_ show that the magnetic interactions in these structures are very similar in strength.

## Conclusions

All four members of the *Ae*_2_FeO_3_Cu*Ch* (*Ae* = Ca, Sr; *Ch* = S, Se) family can be synthesized with high purity using classical high-temperature anaerobic solid-state methods. They crystallize in the *P*4/*nmm* space group in the Sr_2_GaO_3_CuS structure type, where Fe^3+^ cations reside in a square-pyramidal coordination of oxide anions. The Ca_2_FeO_3_CuSe and Ca_2_FeO_3_CuS materials exhibit a structural distortion at low temperatures, as seen using variable temperature XRPD and ED. This involves a √2*a* × √2*a* × *c* expansion of the unit cell (similar to the expanded cell required to explain the magnetic ordering). This subtle distortion can be modeled by a slight buckling of the calcium iron oxide slabs analogous to a tilting distortion of the type found in many perovskite-type phases and is presumably a consequence of the Ca^2+^ ions being slightly small for one or both of their sites. Stable and similar refinements in a model in *P*4̅2*m* were obtained using refinements against both low-temperature synchrotron powder X-ray and powder neutron diffraction (Tables 3 and S3a–e), although we note that the value of the neutron diffraction in locating the light oxide ions is diminished by the overlap of structural superstructure reflections and magnetic reflections, especially at long *d*-spacings.

Variable temperature NPD measurements reveal that at low temperatures, the magnetic models for all four of these compounds consist of antiferromagnetically ordered Fe^3+^ moments tilted away from the crystallographic axes. As the materials are heated, spin reorientations occur toward either the *c*-axis or *ab*-plane. The reorientations of the moments are much more significant for the strontium analogues, where the moment reorientations are complete by ambient temperatures. Due to the lack of unquenched orbital angular momentum in the Fe^3+^ high spin *d* ^5^ ions, the moments have a weak directional preference^33^ and the magnetic structures adopted for the different compounds have subtle differences, which have a temperature dependence. The reason for the differences in the details of the magnetic ordering arrangements and their temperature dependence is not straightforward to rationalize. The comparable values of *T*_N_ for these compounds give evidence that the 180 °Fe–O–Fe superexchange interactions between the Fe^3+^ ions are comparable in magnitude. These results provide key information about the nature of long-range magnetism and spin-reorientations in layered oxide chalcogenides and will help in further understanding of these phenomena.

## Supplementary Material



## Notes

Further tables of refinement parameters, figures showing powder and ED data, and discussion (PDF)
