# Supplementary material for: Structural Distortions and Magnetic Ordering in Ae 2FeO3CuCh (Ae = Ca, Sr; Ch = S, Se) Oxide Chalcogenides
Source: Inorg Chem. 2026 Jul 1;65(28):16505–17. doi: 10.1021/acs.inorgchem.6c02221 (PMC13390028; doi:10.1021/acs.inorgchem.6c02221)
Supplement: Supplementary file 1 [file ic6c02221_si_001.pdf]

Structural distortions and magnetic ordering in Ae_2FeO_3CuCh (*Ae* = Ca, Sr; *Ch* = S, Se) oxide chalcogenides.

Robert D. Smyth,[a†] Bradley C. Sheath,[a†] Lemuel E. Crentsil,[a] Xiaoyu Xu,[a] Simon J. Cassidy,[a] Maria Batuk,[b] Pascal Manuel,[c] Emmanuelle Suard,[d] Andrew N. Fitch,[e] Joke Hadermann[b] and Simon J. Clarke[a,]*

[a]*Department of Chemistry, University of Oxford, Inorganic Chemistry Laboratory, South Parks Road, Oxford OX1 3QR, United Kingdom*

[b]*Electron Microscopy for Materials Science (EMAT), University of Antwerp, B-2020 Antwerp, Belgium*

[c]*ISIS Facility, Rutherford Appleton Laboratory Harwell Oxford, Didcot, OX1 10QX (UK)*

[d]*Institut Laue-Langevin, 71 avenue des Martyrs, CS 20156, 38042 Grenoble Cedex 9, France*

[e] *European Synchrotron Radiation Facility, 71 avenue des Martyrs, CS 40220, 38043 Grenoble Cedex 9, France*

[†]*These authors contributed equally to the work.*

Supporting Information

* Corresponding author

email address: simon.clarke@chem.ox.ac.uk

Table of Contents.

Figure S1. The change in the *c*/*a* ratio for **a)** Sr_2FeO_3CuSe and **b)** Sr_2FeO_3CuS, the normalised change in the *a* and *c* lattice parameter for **c)** Sr_2FeO_3CuSe and **d)** Sr_2FeO_3CuS and the change in volume for **e)** Sr_2FeO_3CuSe and **f)** Sr_2FeO_3CuS as a function of temperature, the error bars are within the data points.

Figure S2. The change in volume for **a)** Ca_2FeO_3CuSe and **b)** Ca_2FeO_3CuS as a function of temperature, the error bars are within the data points.

Figure S3. Room temperature ED patterns of Ca_2FeO_3CuSe. Weak diffuse streaks at (*h*/2 *k*/2 *l*) can be seen in the [110] pattern.

Figure S4. [001] ED pattern acquired at 100 K of Ca_2FeO_3CuSe

Figure S5. Reconstructed [001], [100] and [110] sections at 100 K of Ca_2FeO_3CuSe. Obtained from electron diffraction tomography.

Figure S6. Reconstructed [010], [001] and [100] sections of admixture brownmillerite crystal present in the Ca_2FeO_3CuSe sample at RT.

Figure S7. Electron diffraction patterns at 100 K of Ca_2FeO_3CuS.

Figure S8. Reconstructed [*hk*0] sections at 100 K and RT of Ca_2FeO_3CuS.

Figure S9. Atomic arrangement of the Fe plane viewed along the [001] direction, illustrating the basal expansion of the unit cell with the loss of the *n*-glide in the low temperature $\sqrt{2}a \times \sqrt{2}a \times c$ superstructure. Dark brown circles represent Fe atoms in the upper layer; Orange circles represent Fe atoms in the lower layer.

Figure S10. Magnetisation isotherms collected at room temperature for (a) Ca_2FeO_3CuS, (b) Ca_2FeO_3CuSe, (c) Sr_2FeO_3CuS and (d) Sr_2FeO_3CuSe.

Figure S11. Magnetic susceptibilities of the Ae_2FeO_3CuCh (*Ae* = Ca, Sr; *Ch* = Se, S) family of compounds.

Figure S12. Visual representation of the mM1(a), mM4(a), mA4(a), mA2(a), $m\Gamma_3$ and $m\Gamma_5$ antiferromagnetic modes from which a combination of the two was used to account for the additional magnetic intensity seen in the Ae_2FeO_3CuCh (*Ae* = Ca, Sr; *Ch* = S, Se) family of compounds.

Figure S13. Tilt angle (from the *ab*-plane) of the magnetic moments in Ca_2FeO_3CuSe and Ca_2FeO_3CuS at different temperatures, refined using data collected on the WISH instrument at ISIS. Although the uncertainty on the refined moment increases as the moment becomes smaller on warming, the changes in spin orientation appear to be significant.

Figure S14. PND data obtained for Sr_2FeO_3CuSe on the D2B diffractometer, showing that several magnetic Bragg reflections marked with crosses (+) are present until 400 K.

Figure S15. Rietveld fit for Sr_2FeO_3CuSe measured by D2B diffractometer at 230 K ($\chi 2$ = 2.43, R_{wp} = 6.38), 298 K ($\chi 2$ = 1.99, R_{wp} = 5.27) and 400 K ($\chi 2$ = 2.34, R_{wp} = 6.22)

Figure S16. PND data obtained for Sr_2FeO_3CuS on the WISH diffractometer, the peaks marked with an * are from the mA2(a) magnetic mode contribution and the peaks marked with a + are from the mM4(a) magnetic modes contribution. The + peaks remain above RT.

Figure S17. XRPD pattern measured at 100 K on I11, showing the observed (black), calculated (red) and difference (grey) curves of Sr_2FeO_3CuS, R_{wp}: 4.900 %.

Figure S18. XRPD pattern measured at 100 K on I11, showing the observed (black), calculated (red) and difference (grey) curves Sr_2FeO_3CuSe, R_{wp}: 4.428 %

Table S1a. Comparison of the RT structural model obtained using X-ray and neutron radiation for Ca_2FeO_3CuSe and Ca_2FeO_3CuS. Tables of structural parameters are given in **Tables S1b-S1e**.

Table S1b. Refined atomic parameters from Rietveld refinement of Ca_2FeO_3CuSe at RT using the MAC detector at I11.

Table S1c. Refined atomic parameters from Rietveld refinement of Ca_2FeO_3CuSe at RT using the WISH diffractometer at ISIS.

Table S1d. Refined atomic parameters from Rietveld refinement of Ca_2FeO_3CuS at RT using the MAC detector at I11.

Table S1e. Refined atomic parameters from Rietveld refinement of Ca_2FeO_3CuS at RT using the WISH diffractometer at ISIS.

Table S2a. Comparison of the RT structural model obtained using X-ray and neutron radiation for Sr_2FeO_3CuSe and Sr_2FeO_3CuS. Tables of structural parameters are given in **Tables S2b-S2e**.

Table S2b. Refined atomic parameters from Rietveld refinement of Sr_2FeO_3CuSe at RT using the MAC detector at I11.

Table S2c. Refined atomic parameters from Rietveld refinement of Sr_2FeO_3CuSe at RT using the D2B diffractometer at ISIS.

Table S2d. Refined atomic parameters from Rietveld refinement of Sr_2FeO_3CuS at RT using the MAC detector at I11.

Table S2e. Refined atomic parameters from Rietveld refinement of Sr_2FeO_3CuS at RT using the WISH diffractometer at ISIS.

Table S3a. (similar to Table 3 in the main article). Comparison of the low-T structural model obtained using X-ray and neutron radiation for Ca_2FeO_3CuSe and Ca_2FeO_3CuS. Tables of structural parameters are given in **Tables S3b-S3e**.

Table S3b. Atomic parameters from Rietveld refinement of Ca_2FeO_3CuSe at 4 K, measured using the ID22 diffractometer at the ESRF.

Table S3c. Atomic parameters from Rietveld refinement of Ca_2FeO_3CuSe at 8 K, measured using the WISH diffractometer at ISIS.

Table S3d. Atomic parameters from Rietveld refinement of Ca_2FeO_3CuS at 4 K, measured using the ID22 diffractometer at the ESRF.

Table S3e. Atomic parameters from Rietveld refinement of Ca_2FeO_3CuS at 8 K, measured using the WISH diffractometer at ISIS.

Table S4a. Comparison of the low-T structural model obtained using X-ray and neutron radiation for Sr_2FeO_3CuSe and Sr_2FeO_3CuS. Tables of structural parameters are given in **Tables S3b-S3e**.

Table S4b. Refined atomic parameters from Rietveld refinement of Sr_2FeO_3CuSe at 100 K using the PSD at I11.

Table S4c. Refined atomic parameters from Rietveld refinement of Sr_2FeO_3CuSe at 1.5 K using the WISH diffractometer at ISIS.

Table S4b. Refined atomic parameters from Rietveld refinement of Sr_2FeO_3CuS at 100 K using the PSD at I11.

Table S4e. Refined atomic parameters from Rietveld refinement of Sr_2FeO_3CuS at 1.5 K using the WISH diffractometer at ISIS.

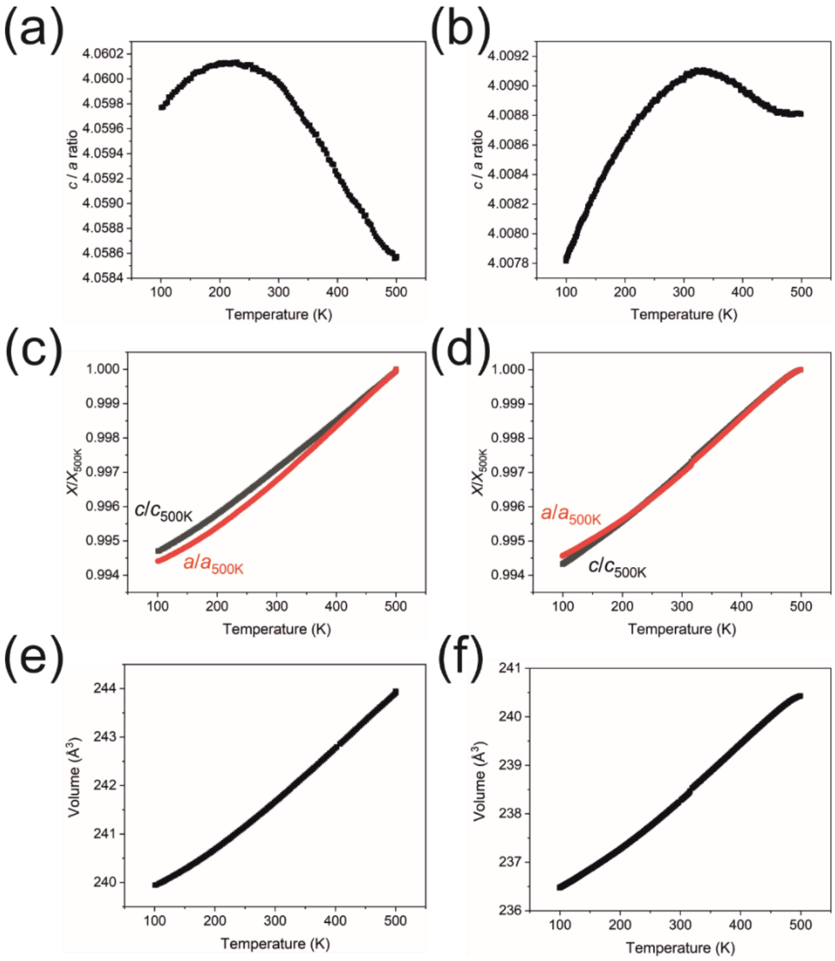

Figure S1. The change in the *c*/*a* ratio for **a)** Sr_2FeO_3CuSe and **b)** Sr_2FeO_3CuS, the normalised change in the *a* and *c* lattice parameter for **c)** Sr_2FeO_3CuSe and **d)** Sr_2FeO_3CuS and the change in volume for **e)** Sr_2FeO_3CuSe and **f)** Sr_2FeO_3CuS as a function of temperature, the error bars are within the data points.

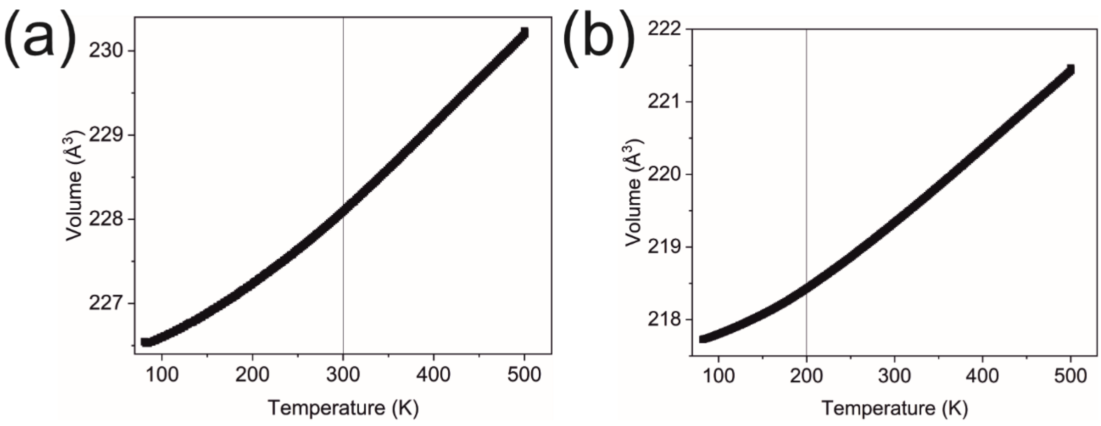

Figure S2. The change in volume for **a)** Ca_2FeO_3CuSe and **b)** Ca_2FeO_3CuS as a function of temperature, the error bars are within the data points.

Electron Diffraction measurements.

Ca_2FeO_3CuSe.

Electron diffraction patterns acquired at room temperature for Ca_2FeO_3CuSe agree with the lattice parameters a = 3.864130 Å and c = 15.31524 Å obtained from powder X-ray diffraction and a reflection condition $h+k$=2n confirmed the *P*4/*nmm* space group. However, weak diffuse streaks at (h/2 k/2 l) were evident in the [110] patterns as shown in Figure 4 of the main article. Note that these were not observed for the Ca_2FeO_3CuS sample.

The sample of Ca_2FeO_3CuSe was cooled down in-situ to 100 K. The diffuse streaks present in the room temperature [110] pattern become distinct extra reflections in what becomes the [100] ED pattern for the supercell (see Figure 4 of the main article). These extra reflections confirm the transformation to the $\sqrt{2}a \times \sqrt{2}a \times c$ cell. Due to constraints on the sample holder, it was not possible to orient the crystal perfectly along the [001] direction. Nevertheless, the ED pattern shown in Figure S4 confirms the appearance of new reflections compared with the room temperature diffraction pattern in Figure S3. 3D ED data were acquired at 100K for a crystal of a size around 200 nm using a dedicated tomography holder. The tilting range was –56 to +75 degrees with a step of 1°. The reconstructed [001], [100] and [110] zones obtained using this method are shown in Figure S5, they match to the directly measured in-zone SAED patterns. The patterns demonstrate no reflection conditions, indicating the extinction symbol *P*---.

An admixture crystal containing only Ca, Fe and O (Ca and Fe in equal amounts) was found. It corresponds to $Ca_2Fe_2O_5$ brownmillerite with *Pnma* structure and a = 5.4 Å, b = 14.8 Å, c = 5.4 Å, and was incorporated into the refinements against the powder diffraction data (main article). The reconstructed [010], [001] and [100] zones for this $Ca_2Fe_2O_5$ side phase are shown in Figure S6.

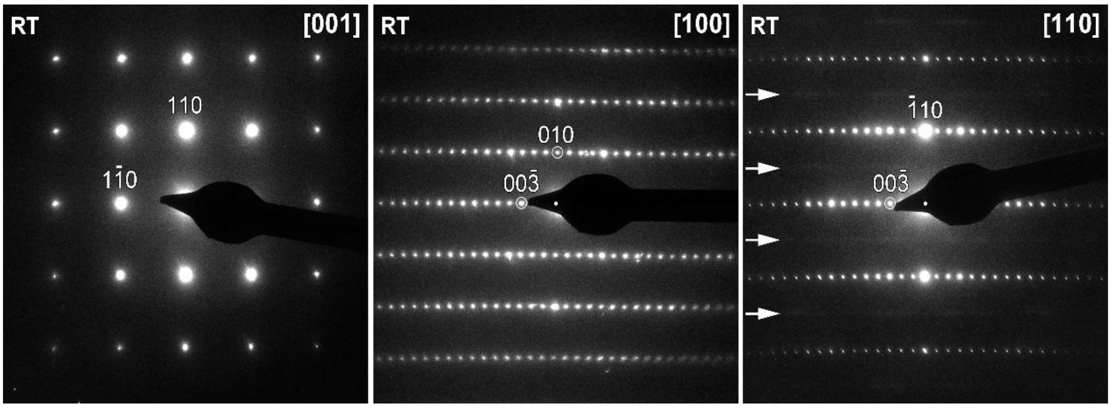

Figure S3. Room temperature ED patterns of Ca_2FeO_3CuSe. Weak diffuse streaks at (h/2 k/2 l) can be seen in the [110] pattern. These transform into distinct peaks as shown in Figure 4 of the main article.

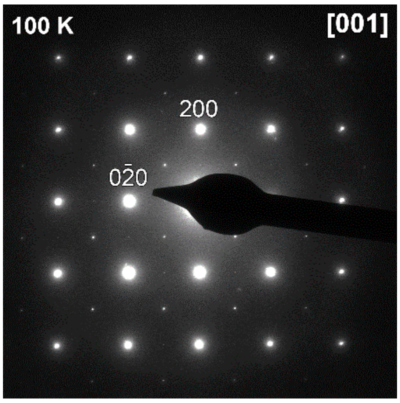

Figure S4. [001] ED pattern acquired at 100 K of Ca_2FeO_3CuSe. New reflections compared with the room temperature pattern in Figure S3 confirming the $\sqrt{2}a \times \sqrt{2}a \times c$ cell expansion.

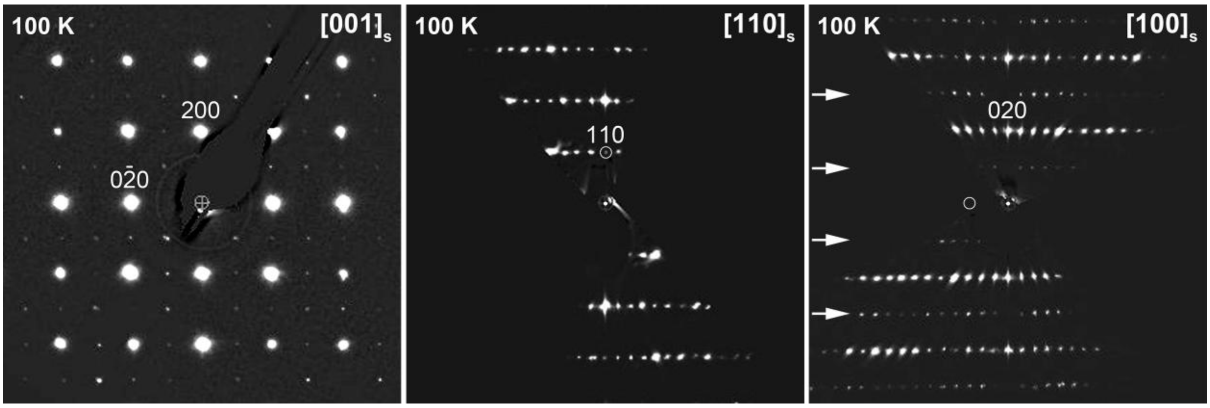

Figure S5. Reconstructed [001], [100] and [110] sections at 100 K of Ca_2FeO_3CuSe. Obtained from 3D ED measurements.

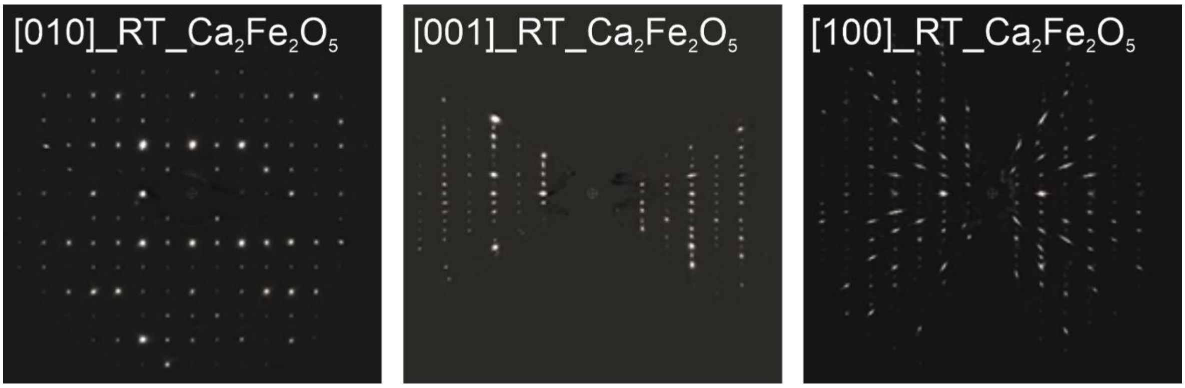

Figure S6. Reconstructed [010], [001] and [100] sections of admixture brownmillerite crystal $Ca_2Fe_2O_5$ present in the Ca_2FeO_3CuSe sample at RT. Obtained from electron diffraction tomography.

Ca_2FeO_3CuS.

Comparative room temperature and 100 K electron diffraction patterns are shown in Figure 9 of the main text. Additional electron diffraction patters are shown below.

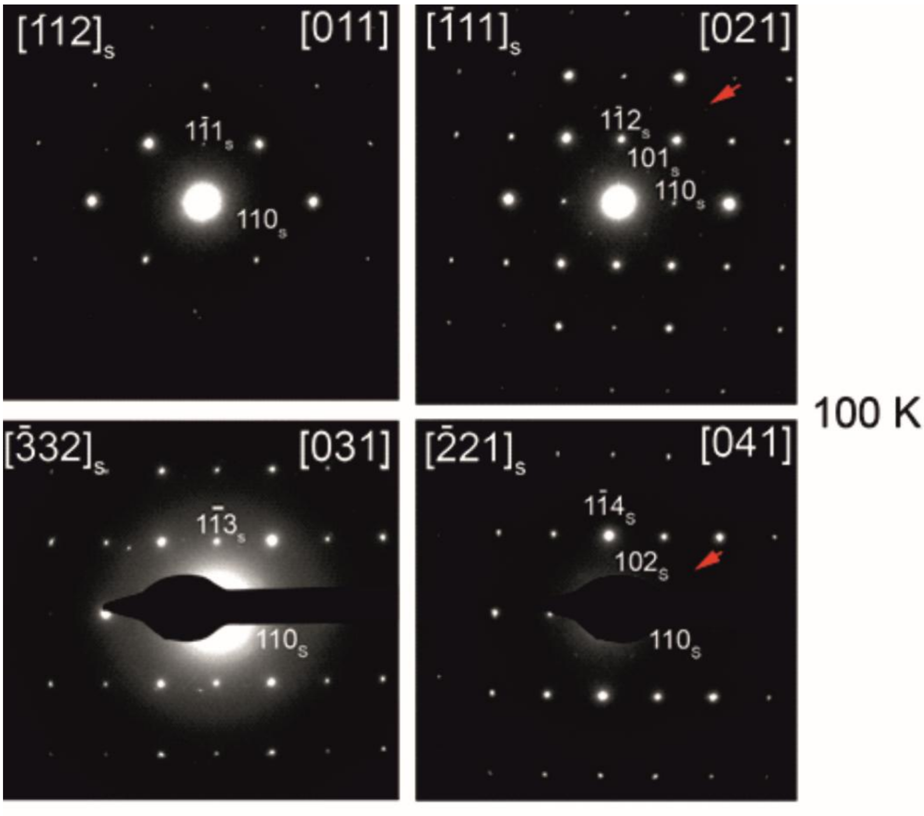

Figure S7. Electron diffraction patterns for Ca_2FeO_3CuS at 100 K showing [0*kl*] zone reflections (referred to the subcell indexing appropriate to room temperature). The subscript “s” identifies these zones and the reflections indexed according to the low temperature $\sqrt{2}a \times \sqrt{2}a \times c$ supercell.

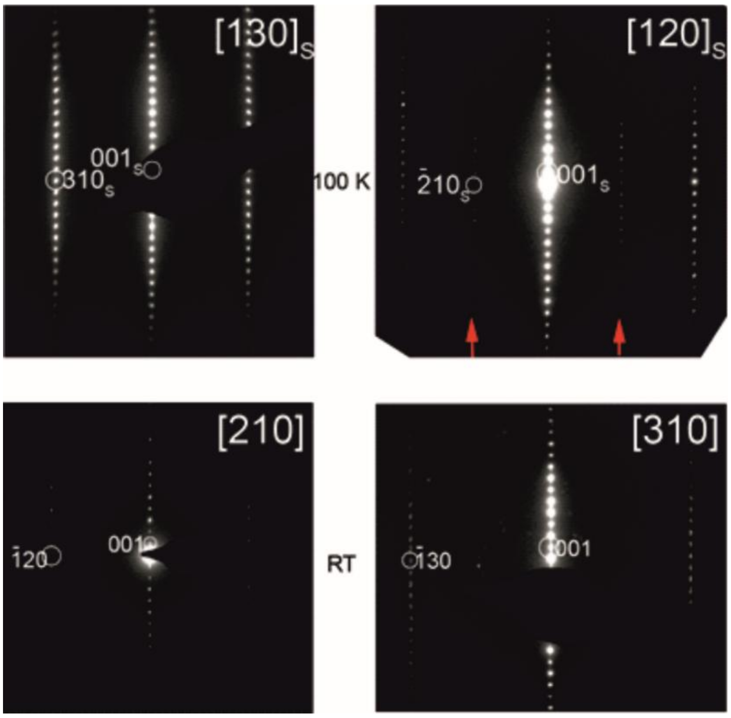

Figure S8. Electron diffraction patterns for [*hk*0] zones at 100 K and RT for Ca_2FeO_3CuS. Subscript "s" indicates the √2*a* × √2*a* × *c* supercell indexing.

The *hk*0: *h* + *k* = 2*n* reflection condition was suggested for Ca_2FeO_3CuS from electron diffraction patterns in the [001] zone. Figure S9 shows the Fe atoms in the two layers of basal-vertex-linked FeO_5 square-based pyramids. The two layers are related by the *n*-glide plane in the room temperature structure in space group *P*4/*nmm* (dashed lines show the unit cell). The Fe coordinates for the high temperature structure using this subcell are ½ 0 *z* and 0 ½ -*z* for the lighter and darker circles (2*c* position using origin choice 1 for *P*4/*nmm*) and are related by the *n* glide. If the low temperature √2*a* × √2*a* × *c* supercell is overlaid (continuous lines) on to the atomic arrangement, it can be seen that an *n*-glide perpendicular to the *c* axis does not relate atoms in the two different layers, and, in the expanded cell, does not in general relate pairs of like atoms in the structure that do not have *z* = 0 or ½. Similar considerations apply to other space groups in which there is an *n*-glide such as *Pmmn.* Since the electron diffraction patterns for Ca_2FeO_3CuSe show only weak superstructure reflections and since the other diffraction data suggest similar models, we propose that the *hk*0: *h* + *k* ≠ 2*n* reflections in Ca_2FeO_3CuS are too weak to be observed.

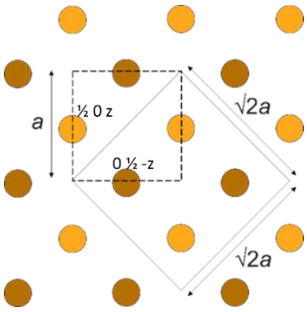

Figure S9. Atomic arrangement of the Fe ions in the double layer of FeO_5 basal-vertex-shared polyhedra viewed along the [001] direction, illustrating the basal expansion of the unit cell with the loss of the *n*-glide in the low temperature √2*a* × √2*a* × *c* superstructure. Dark brown circles represent Fe atoms in the upper layer; Orange circles represent Fe atoms in the lower layer.

Magnetic ordering.

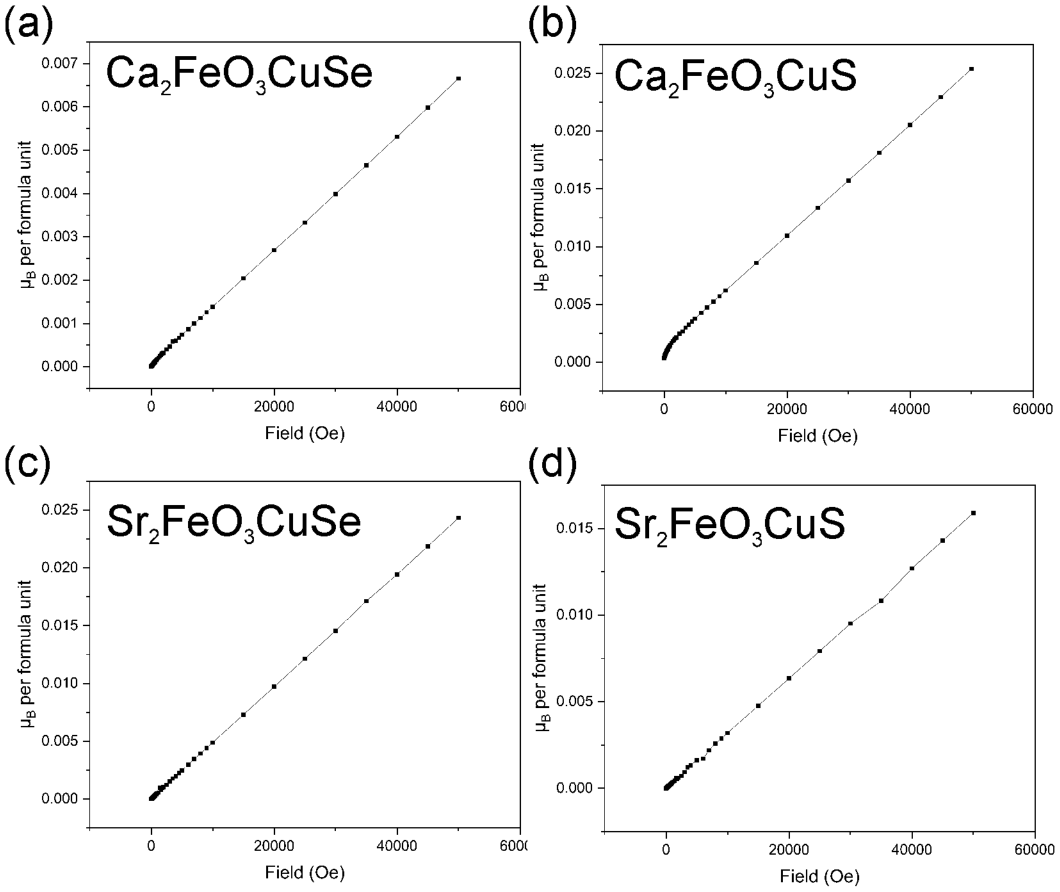

Figure S10. Magnetisation isotherms collected at room temperature (below the long-range magnetic ordering transitions for (a) Ca_2FeO_3CuSe, (b) Ca_2FeO_3CuS, (c) Sr_2FeO_3CuSe and (d) Sr_2FeO_3CuS. The low-field curvature in the case of Ca_2FeO_3CuS (b) is likely due to a minuscule magnetite impurity (see Figure S11).

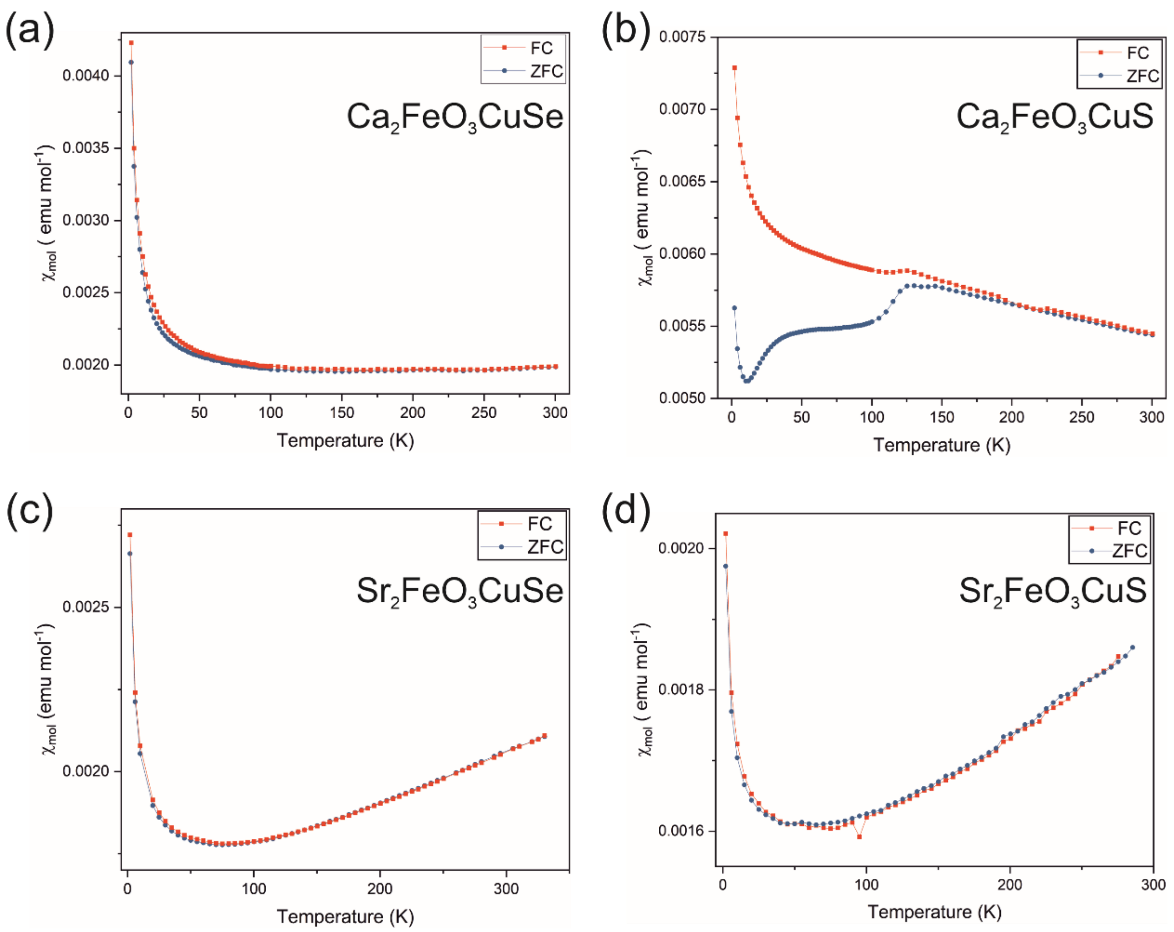

Figure S11. Magnetic susceptibilities of the Ae_2FeO_3CuCh (*Ae* = Ca, Sr; *Ch* = Se, S) family of compounds. The data are rather uninformative because these antiferromagnets are ordered above room temperature according to the neutron powder data, and just show small Curie tails from minuscule numbers of impurity spins at the lowest temperatures. The feature at around 120 K in the Ca_2FeO_3CuS sample likely indicates a minuscule amount of magnetite below the detection limits of the diffraction techniques and is consistent with the low-field curvature of the isotherm in Figure S10(b).

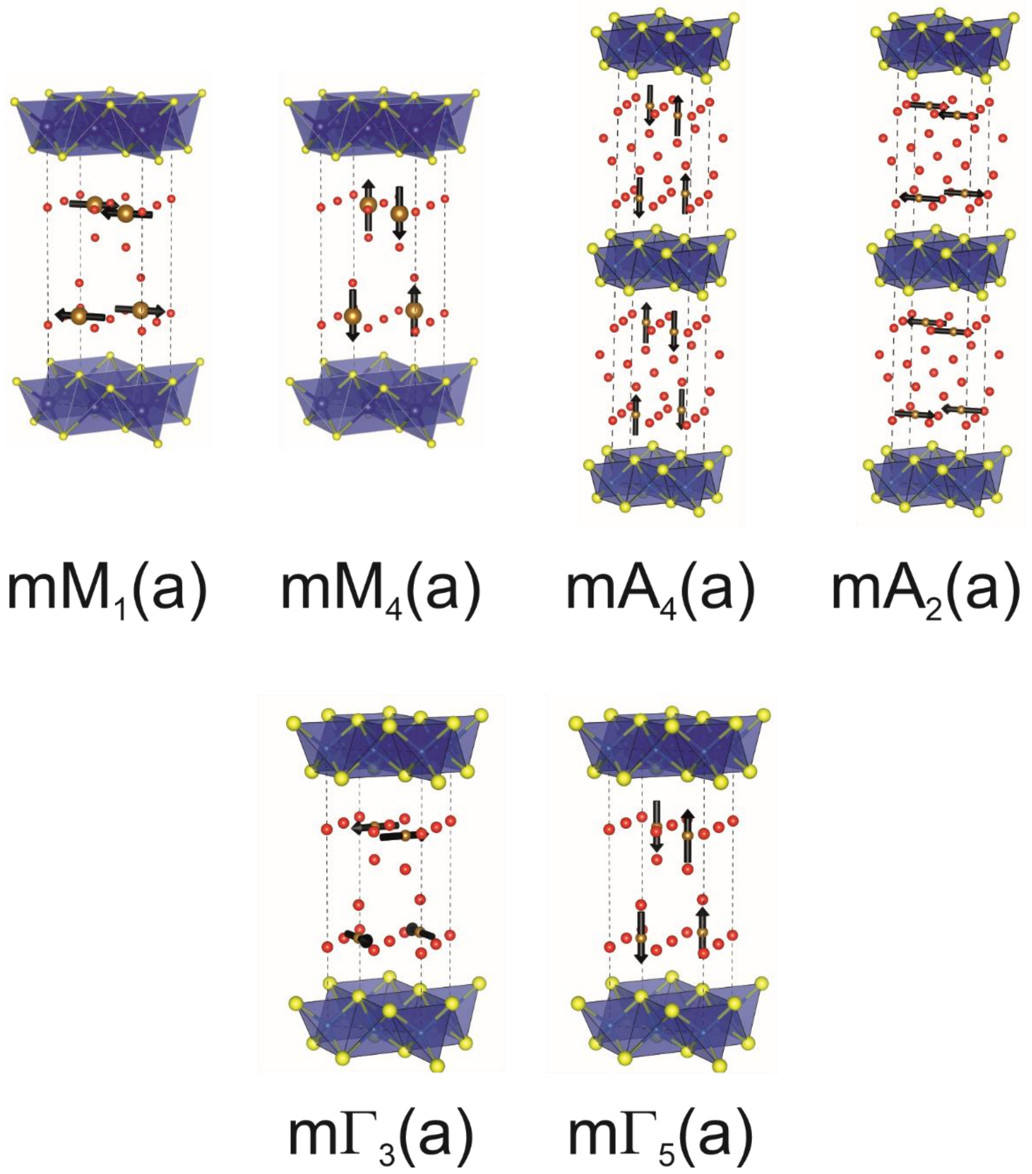

Figure S12. Visual representation of the mM1(a), mM4(a), mA4(a), mA2(a), $m\Gamma_3$ and $m\Gamma_5$ antiferromagnetic modes which were used in combinations listed in Table 3 of the main article to account for magnetic Bragg intensity seen in the Ae_2FeO_3CuCh (*Ae* = Ca, Sr; *Ch* = S, Se) family of compounds.

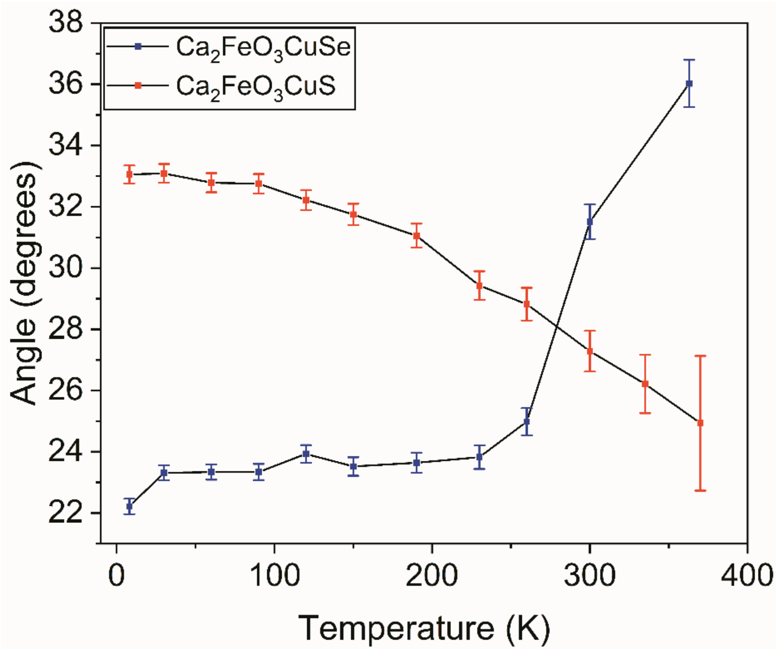

Figure S13. Tilt angle (from the *ab*-plane) of the magnetic moments in Ca_2FeO_3CuSe and Ca_2FeO_3CuS at different temperatures, refined using data collected on the WISH instrument at ISIS. Although the uncertainty on the refined moment increases as the moment becomes smaller on warming, the changes in spin orientation appear to be significant.

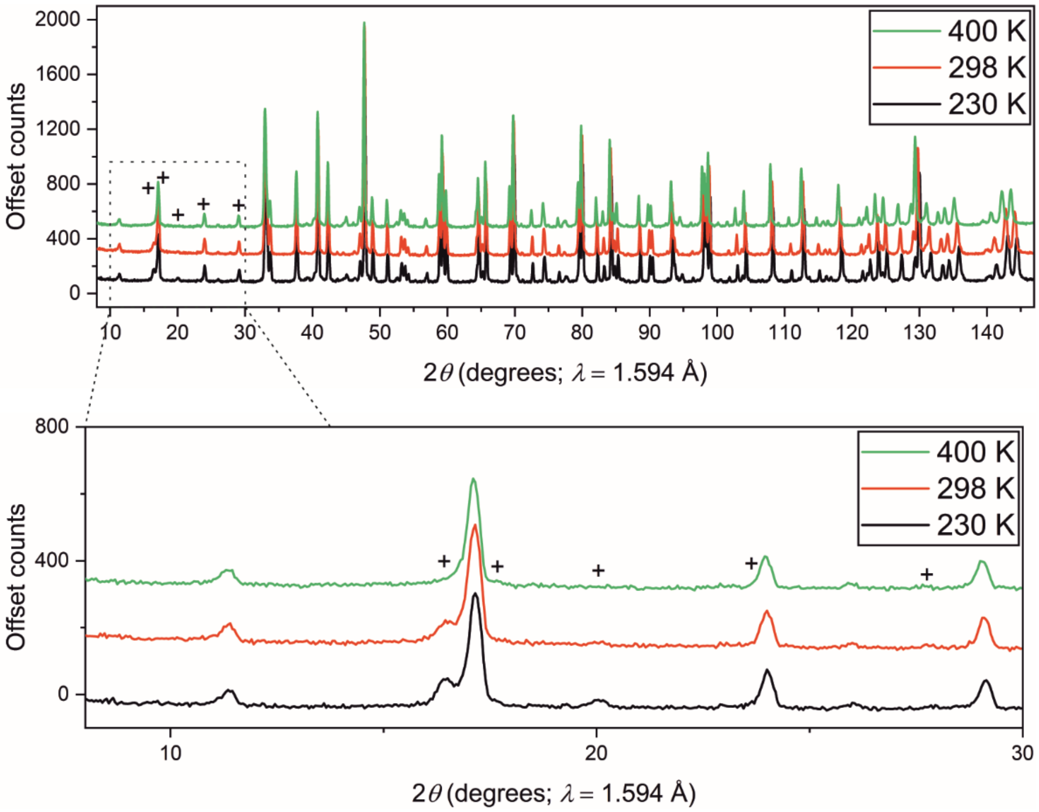

Figure S14. PND data obtained for Sr_2FeO_3CuSe on the D2B diffractometer, showing that several magnetic Bragg reflections marked with crosses (+) are present at 298 K, but not 400 K. (See also Figure 19 of the main article).

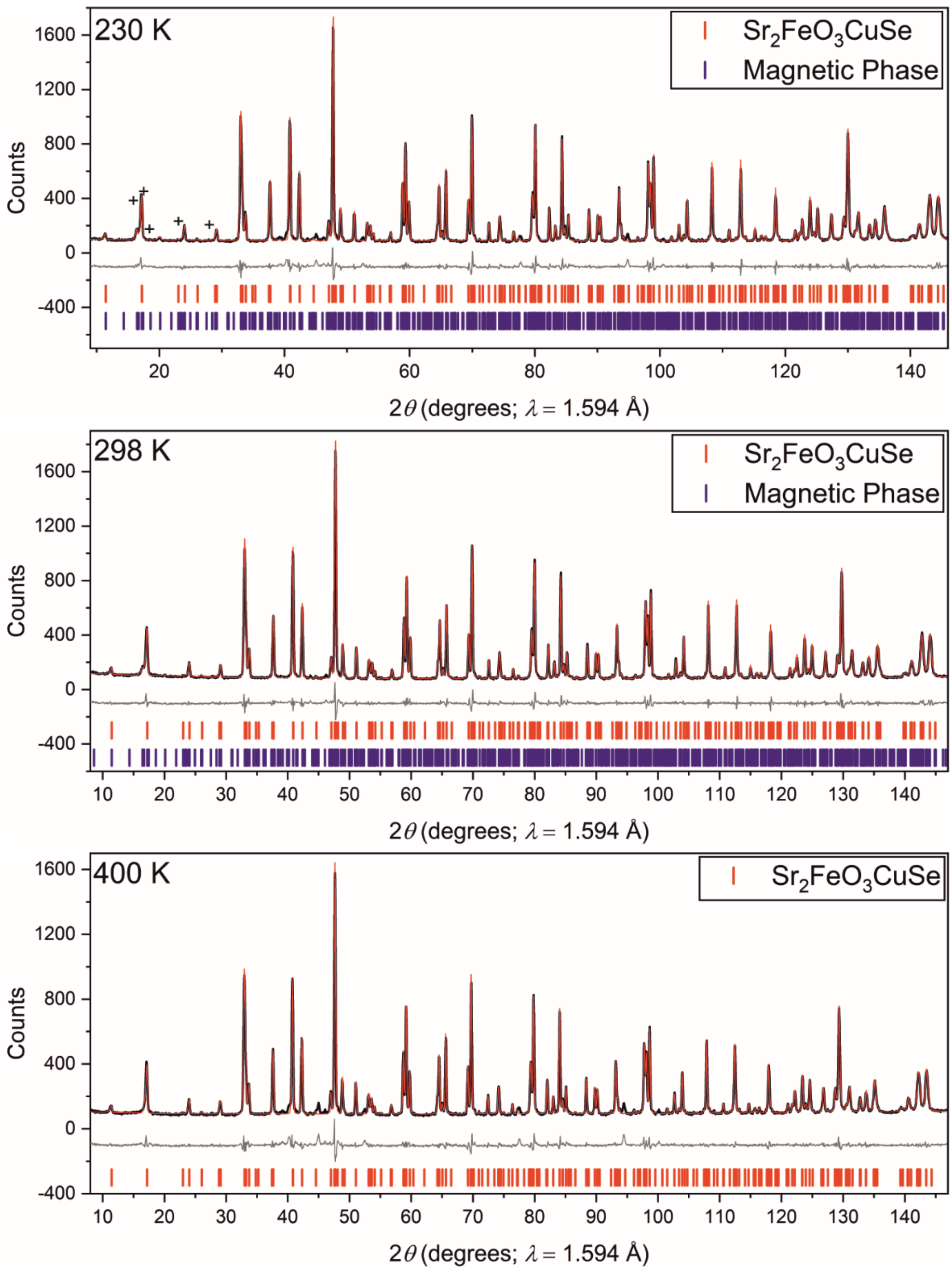

Figure S15. Rietveld fits for Sr_2FeO_3CuSe measured by D2B diffractometer at 230 K ($\chi2$ = 2.43, R_{wp} = 6.38), 298 K ($\chi2$ = 1.99, R_{wp} = 5.27) and 400 K ($\chi2$ = 2.34, R_{wp} = 6.22)

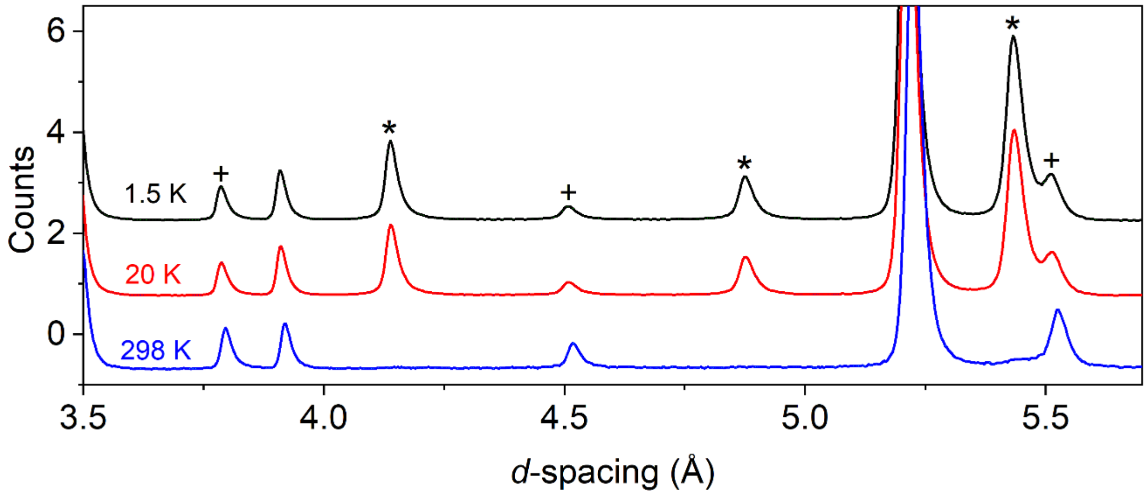

Figure S16. PND data obtained for Sr_2FeO_3CuS on the WISH diffractometer, the peaks marked with an * are from the mA2(a) magnetic mode contribution and the peaks marked with a + are from the mM4(a) magnetic mode contribution. The + peaks remain above RT.

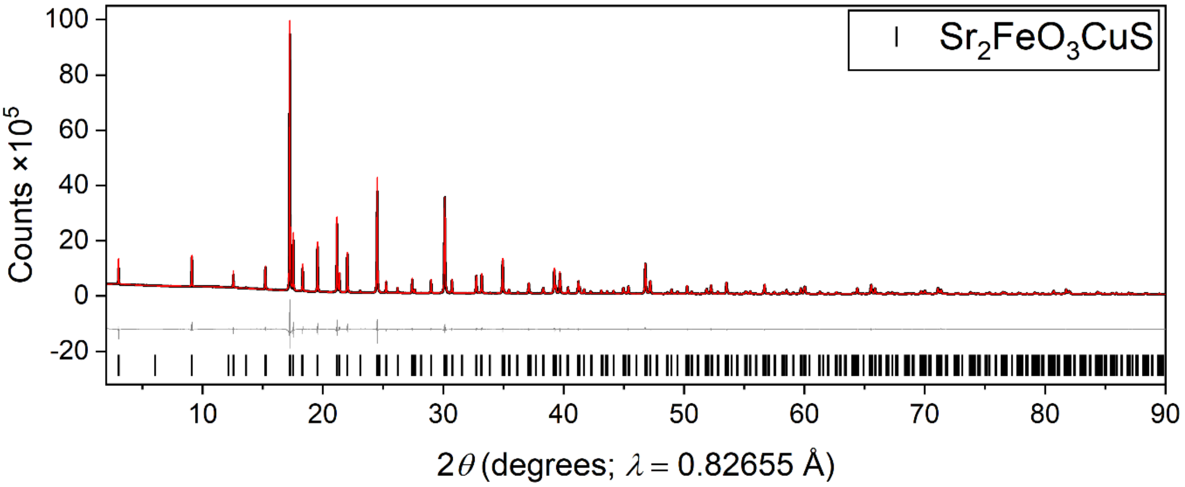

Figure S17. XRPD pattern measured at 100 K on I11, showing the observed (black), calculated (red) and difference (grey) curves of Sr_2FeO_3CuS, R_{wp}: 4.900 %.

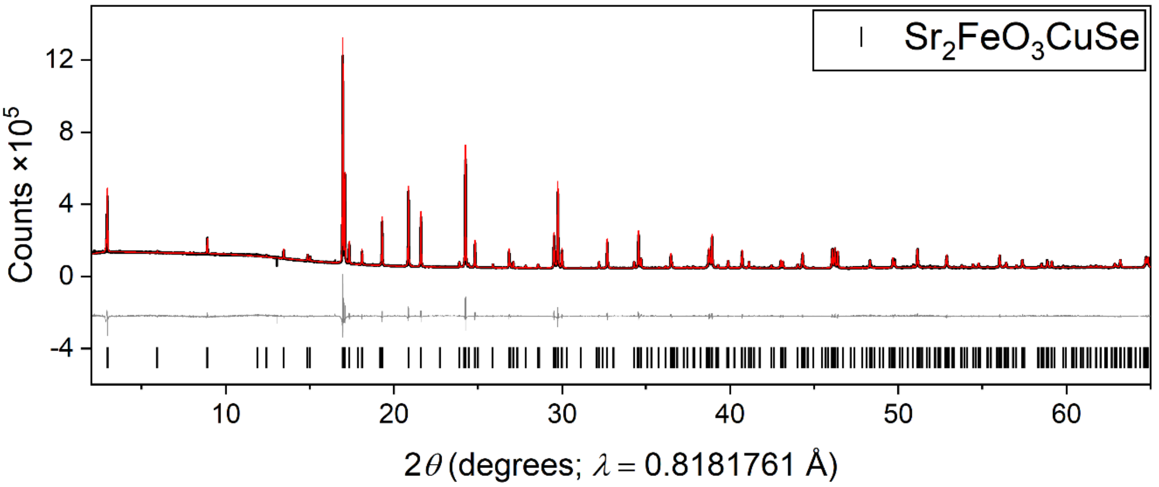

Figure S18. XRPD pattern measured at 100 K on I11, showing the observed (black), calculated (red) and difference (grey) curves Sr_2FeO_3CuSe, R_{wp}: 4.428 %. The dip in intensity below background at 13° is an instrumental artefact.

Table S1a. Comparison of the RT structural model obtained using X-ray and neutron radiation for Ca_2FeO_3CuSe and Ca_2FeO_3CuS. Tables of structural parameters are given in **Tables S1b-S1e**.

	Ca_2FeO_3CuSe		Ca_2FeO_3CuS	
Radiation type	X-ray (I11)	Neutron (WISH)	X-ray (I11)	Neutron (WISH)
Temperature (K)	300	300	300	300
Space Group	$P4/nmm$	$P4/nmm$	$P4/nmm$	$P4/nmm$
R_{wp} (%)	8.040	9.891	8.407	4.249
χ^2	4.166	0.149	4.202	0.056
a (Å)	3.864130(5)	3.86828(2)	3.829500(11)	3.83318(6)
c (Å)	15.31524(3)	15.3334(1)	14.95914(7)	14.9767(3)
V (Å^3)	228.679(1)	229.442(3)	219.377(2)	220.056(8)
Fe-*Ch* (Å) [1]	3.2357(8)	3.240(3)	3.1649(16)	3.186(3)
Fe-O(1) (Å) [4]	1.9596(4)	1.9584(3)	1.9434(4)	1.9420(2)
Fe-O(2) (Å) [1]	1.843(3)	1.844(3)	1.888(4)	1.887(2)

Table S1b. Refined atomic parameters from Rietveld refinement of Ca_2FeO_3CuSe at RT using the MAC detector at I11.

Atom	Site	*x*	*y*	*z*	Occ	B_{eq} (Å^2)
Ca1	2*c*	0.75	0.75	0.19825(6)	1	1.66(1)
Ca2	2*c*	0.75	0.75	0.41606(5)	1	2.24(2)
Fe1	2*c*	0.25	0.25	0.31574(4)	1	1.51(1)
Cu1	2*a*	0.25	0.75	0	1	2.46(1)
Se1	2*c*	0.25	0.25	0.10447(3)	1	1.75(9)
O1	4*f*	0.25	0.75	0.2944(1)	1	1.81(4)
O2	2*c*	0.25	0.25	0.4361(2)	1	2.60(6)

Table S1c. Refined atomic parameters from Rietveld refinement of Ca_2FeO_3CuSe at RT using the WISH diffractometer at ISIS.

Atom	Site	*x*	*y*	*z*	Occ	B_{eq} (Å^2)
Ca1	2*c*	0.75	0.75	0.1993(1)	1	1.07(5)
Ca2	2*c*	0.75	0.75	0.41730(9)	1	1.34(8)
Fe1	2*c*	0.25	0.25	0.31575(6)	1	0.98(6)
Cu1	2*a*	0.25	0.75	0	1	1.31(6)
Se1	2*c*	0.25	0.25	0.10522(7)	1	3.15(9)
O1	4*f*	0.25	0.75	0.29491(7)	1	2.04(7)
O2	2*c*	0.25	0.25	0.4365(1)	1	2.16(6)

Table S1d. Refined atomic parameters from Rietveld refinement of Ca_2FeO_3CuS at RT using the MAC detector at I11.

Atom	Site	*x*	*y*	*z*	Occ	B_{eq} (Å^2)
Ca1	2*c*	0.75	0.75	0.18989(9)	1	0.97(2)
Ca2	2*c*	0.75	0.75	0.41403(7)	1	1.27(3)
Fe1	2*c*	0.25	0.25	0.31028(5)	1	0.72(2)
Cu1	2*a*	0.25	0.75	0	1	1.70(2)
S1	2*c*	0.25	0.25	0.09860(9)	1	1.10(3)
O1	4*f*	0.25	0.75	0.2885(1)	1	1.21(5)
O2	2*c*	0.25	0.25	0.4356(2)	1	2.05(1)

Table S1e. Refined atomic parameters from Rietveld refinement of Ca_2FeO_3CuS at RT using the WISH diffractometer at ISIS.

Atom	Site	*x*	*y*	*z*	Occ	B_{eq} (Å^2)
Ca1	2*c*	0.75	0.75	0.1896(1)	1	0.60(6)
Ca2	2*c*	0.75	0.75	0.4133(1)	1	2.20(9)
Fe1	2*c*	0.25	0.25	0.30946(5)	1	0.45(3)
Cu1	2*a*	0.25	0.75	0	1	1.46(5)
S1	2*c*	0.25	0.25	0.0967(1)	1	1.30(1)
O1	4*f*	0.25	0.75	0.28819(4)	1	1.10(6)
O2	2*c*	0.25	0.25	0.4355(1)	1	2.56(1)

Table S2a. Comparison of the RT structural model obtained using X-ray and neutron radiation for Sr_2FeO_3CuSe and Sr_2FeO_3CuS. Tables of structural parameters are given in **Tables S2b-S2e**.

	Sr_2FeO_3CuSe		Sr_2FeO_3CuS	
Radiation type	X-ray (I11)	Neutron (D2B)	X-ray (I11)	Neutron (WISH)
Temperature (K)	300	300	300	300
Space Group	$P4/nmm$	$P4/nmm$	$P4/nmm$	$P4/nmm$
R_{wp} (%)	7.007	5.446	5.660	4.889
χ^2	4.488	2.051	3.267	0.052
a (Å)	3.937591(6)	3.93702(2)	3.901953(5)	3.90596(3)
c (Å)	15.97075(3)	15.9685(1)	15.643879(3)	15.6602(2)
V (Å^3)	247.620(1)	247.513(3)	238.182(1)	238.920(5)
Fe-*Ch* (Å) [1]	3.4609(8)	3.459(3)	3.4082(13)	3.416(1)
Fe-O(1) (Å) [4]	1.9956(4)	1.9956(4)	1.9779(2)	1.9831(3)
Fe-O(2) (Å) [1]	1.873(4)	1.889(3)	1.893(2)	1.896(2)

Table S2b. Refined atomic parameters from Rietveld refinement of Sr_2FeO_3CuSe at RT using the MAC detector at I11.

Atom	Site	*x*	*y*	*z*	Occ	B_{eq} (Å^2)
Sr1	2*c*	0.75	0.75	0.19216(4)	1	0.308(1)
Sr2	2*c*	0.75	0.75	0.41368(3)	1	0.40(1)
Fe1	2*c*	0.25	0.25	0.31364(5)	1	0.26(1)
Cu1	2*a*	0.25	0.75	0	1	1.07(1)
Se1	2*c*	0.25	0.25	0.09730(3)	1	0.39(1)
O1	4*f*	0.25	0.75	0.2912(1)	1	0.50(4)
O2	2*c*	0.25	0.25	0.4308(2)	1	0.62(6)

Table S2c. Refined atomic parameters from Rietveld refinement of Sr_2FeO_3CuSe at RT using the D2B diffractometer at ISIS.

Atom	Site	*x*	*y*	*z*	Occ	B_{eq} (Å^2)
Sr1	2*c*	0.75	0.75	0.1924(1)	1	0.29(3)
Sr2	2*c*	0.75	0.75	0.4137(1)	1	0.42(3)
Fe1	2*c*	0.25	0.25	0.31431(9)	1	0.16(2)
Cu1	2*a*	0.25	0.75	0	1	1.02(3)
Se1	2*c*	0.25	0.25	0.0977(1)	1	0.40(4)
O1	4*f*	0.25	0.75	0.2938(1)	1	0.40(3)
O2	2*c*	0.25	0.25	0.4327(2)	1	0.62(4)

Table S2d. Refined atomic parameters from Rietveld refinement of Sr_2FeO_3CuS at RT using the MAC detector at I11.

Atom	Site	*x*	*y*	*z*	Occ	B_{eq} (Å^2)
Sr1	2*c*	0.75	0.75	0.18448(2)	1	0.334(7)
Sr2	2*c*	0.75	0.75	0.41219(2)	1	0.405(8)
Fe1	2*c*	0.25	0.25	0.31027(3)	1	0.164(8)
Cu1	2*a*	0.25	0.75	0	1	1.17(1)
S1	2*c*	0.25	0.25	0.09210(6)	1	0.68(2)
O1	4*f*	0.25	0.75	0.29004(6)	1	0.34(3)
O2	2*c*	0.25	0.25	0.4315(1)	1	0.43(4)

Table S2e. Refined atomic parameters from Rietveld refinement of Sr_2FeO_3CuS at RT using the WISH diffractometer at ISIS.

Atom	Site	*x*	*y*	*z*	Occ	B_{eq} (Å^2)
Sr1	2*c*	0.75	0.75	0.1845(1)	1	0.426(4)
Sr2	2*c*	0.75	0.75	0.41293(8)	1	0.441(3)
Fe1	2*c*	0.25	0.25	0.30951(7)	1	0.408(2)
Cu1	2*a*	0.25	0.75	0	1	1.07(5)
S1	2*c*	0.25	0.25	0.0934(2)	1	1.19(4)
O1	4*f*	0.25	0.75	0.28751(5)	1	0.77(7)
O2	2*c*	0.25	0.25	0.43061(9)	1	0.983(2)

Table S3a. (similar to Table 3 in the main article). Comparison of the low-T structural model obtained using X-ray and neutron radiation for Ca_2FeO_3CuSe and Ca_2FeO_3CuS. Tables of structural parameters are given in **Tables S3b-S3e**.

	Ca_2FeO_3CuSe		Ca_2FeO_3CuS	
Radiation type	X-ray (ESRF)	Neutron (WISH)	X-ray (ESRF)	Neutron (WISH)
Temperature (K)	4	8	4	8
Space Group	$P\bar{4}2m$	$P\bar{4}2m$	$P\bar{4}2m$	$P\bar{4}2m$
R_{wp} (%)	9.52	7.54	5.41	3.67
χ^2	3.22	0.10	1.60	0.05
a (Å)	5.458390(8)	5.46404(3)	5.407963(7)	5.41141(8)
c (Å)	15.22527(4)	15.2466(1)	14.88044(3)	14.8955(3)
V (Å^3)	453.612(2)	455.199(6)	435.194(2)	436.19(2)
Fe-*Ch* (Å) [1]	3.2142(6)	3.196(4)	3.1461(7)	3.100(5)
Fe-O(1) (Å) [2]	1.966(9)	1.964(2)	1.946(2)	1.939(2)
Fe-O(2) (Å) [1]	2.003(2)	1.994(7)	1.986(2)	2.004(6)
Fe-O(3) (Å) [1]	1.896(3)	1.964(5)	1.885(2)	1.902(6)
Fe-O(4) (Å) [1]	1.911(2)	1.906(5)	1.908(2)	1.899(4)

Table S3b. Atomic parameters from Rietveld refinement of Ca_2FeO_3CuSe at 4 K, measured using the ID22 diffractometer at the ESRF.

Atom	Site	*x*	*y*	*z*	B_{eq} (Å^2)
Se	4*n*	0.7508(1)	0.7508(1)	0.89545(2)	0.141(6)
Cu1	1*a*	0	0	0	0.8(1)
Cu2	2*e*	0.5	0	0	0.22(8)
Cu3	1*d*	0.5	0.5	0	0.25(7)
Fe1	4*n*	0.2575(2)	0.2575(2)	0.68439(2)	0.039(8)
Ca1	4*n*	0.2489(3)	0.7511(3)	0.80190(4)	0.202(9)
Ca2	4*n*	0.2456(3)	0.7544(4)	0.58394(4)	0.60(1)
O1	4*m*	0.5	0	0.7087(7)	0.34(2)
O2	2*g*	0	0	0.6989(7)	0.34(2)
O3	2*h*	0.5	0.5	0.706(1)	0.34(2)
O4	4*n*	0.7813(4)	0.2187(4)	0.4396(2)	0.34(2)

Table S3c. Atomic parameters from Rietveld refinement of Ca_2FeO_3CuSe at 8 K, measured using the WISH diffractometer at ISIS.

Atom	Site	*x*	*y*	*z*	B_{eq} (Å^2)
Se	4*n*	0.7586(7)	0.7586(7)	0.8948(3)	0.4(1)
Cu1	1*a*	0	0	0	0.51(5)
Cu2	2*e*	0.5	0	0	0.5(2)
Cu3	1*d*	0.5	0.5	0	2.1(6)
Fe1	4*n*	0.2460(6)	0.2460(6)	0.6845(3)	0.55(6)
Ca1	4*n*	0.2271(9)	0.7730(9)	0.8050(6)	0.02(1)
Ca2	4*n*	0.234(1)	0.765(1)	0.5806(2)	0.64(1)
O1	4*m*	0.5	0	0.7078(5)	1.7(1)
O2	2*g*	0	0	0.726(1)	0.1(3)
O3	2*h*	0.5	0.5	0.6878(8)	2.1(4)
O4	4*n*	0.7780(8)	0.2220(8)	0.4416(4)	0.4(1)

Table S3d. Atomic parameters from Rietveld refinement of Ca_2FeO_3CuS at 4 K, measured using the ID22 diffractometer at the ESRF.

Atom	Site	*x*	*y*	*z*	B_{eq} (Å^2)
Se	4*n*	0.7546(3)	0.7546(3)	0.90160(4)	0.21(1)
Cu1	1*a*	0	0	0	0.66(5)
Cu2	2*e*	0.5	0	0	0.39(3)
Cu3	1*d*	0.5	0.5	0	0.43(5)
Fe1	4*n*	0.2567(1)	0.2567(1)	0.69026(2)	0.028(9)
Ca1	4*n*	0.2468(2)	0.7532(2)	0.81067(3)	0.19(1)
Ca2	4*n*	0.2423(2)	0.7577(2)	0.58630(3)	0.35(1)
O1	4*m*	0.5	0	0.7143(5)	0.25(2)
O2	2*g*	0	0	0.7102(7)	0.25(2)
O3	2*h*	0.5	0.5	0.7104(7)	0.25(2)
O4	4*n*	0.7739(4)	0.2261(4)	0.4370(1)	0.25(2)

Table S3e. Atomic parameters from Rietveld refinement of Ca_2FeO_3CuS at 8 K, measured using the WISH diffractometer at ISIS.

Atom	Site	*x*	*y*	*z*	B_{eq} (Å^2)
Se	4*n*	0.720(1)	0.720(1)	0.8978(3)	0.89(2)
Cu1	1*a*	0	0	0	2.1(3)
Cu2	2*e*	0.5	0	0	0.6(1)
Cu3	1*d*	0.5	0.5	0	1.1(3)
Fe1	4*n*	0.2539(7)	0.2539(7)	0.6900(1)	0.07(4)
Ca1	4*n*	0.248(1)	0.752(1)	0.8116(2)	0.23(6)
Ca2	4*n*	0.246(1)	0.754(1)	0.5841(2)	0.09(6)
O1	4*m*	0.5	0	0.7113(6)	0.7(1)
O2	2*g*	0	0	0.7231(8)	0.7(3)
O3	2*h*	0.5	0.5	0.7078(8)	0.6(1)
O4	4*n*	0.771(1)	0.229(1)	0.437(1)	1.00(9)

Table S4a. Comparison of the low-T structural model obtained using X-ray and neutron radiation for Sr_2FeO_3CuSe and Sr_2FeO_3CuS. Tables of structural parameters are given in **Tables S3b-S3e**.

	Sr_2FeO_3CuSe		Sr_2FeO_3CuS	
Radiation type	X-ray (I11)	Neutron (WISH)	X-ray (I11)	Neutron (WISH)
Temperature (K)	100 K	1.5 K	100 K	1.5
Space Group	$P4/nmm$	$P4/nmm$	$P4/nmm$	$P4/nmm$
R_{wp} (%)	4.43	6.08	4.90	7.78
χ^2	3.75	0.05	6.41	0.10
a (Å)	3.89525(1)	3.9289(5)	3.892199(6)	3.89604(8)
c (Å)	15.81382(8)	15.9354(2)	15.59916(4)	15.6159(4)
V (Å^3)	239.943(2)	245.99(7)	236.315(1)	237.04(1)
Fe-*Ch* (Å) [1]	3.429(3)	3.448(4)	3.394(2)	3.400(7)
Fe-O(1) (Å) [4]	1.968(1)	1.9922(7)	1.9703(4)	1.9770(6)
Fe-O(2) (Å) [1]	1.907(7)	1.896(5)	1.886(4)	1.905(4)

Table S4b. Refined atomic parameters from Rietveld refinement of Sr_2FeO_3CuSe at 100 K using the PSD at I11.

Atom	Site	*x*	*y*	*z*	Occ	B_{eq} (Å^2)
Sr1	2*c*	0.75	0.75	0.1925(1)	1	0.24(3)
Sr2	2*c*	0.75	0.75	0.41334(9)	1	033(3)
Fe1	2*c*	0.25	0.25	0.3143(1)	1	0.47(4)
Cu1	2*a*	0.25	0.75	0	1	0.11(3)
Se1	2*c*	0.25	0.25	0.09747(9)	1	0.20(3)
O1	4*f*	0.25	0.75	0.2966(4)	1	0.34(1)
O2	2*c*	0.25	0.25	0.4349(4)	1	0.69(1)

Table S4c. Refined atomic parameters from Rietveld refinement of Sr_2FeO_3CuSe at 1.5 K using the WISH diffractometer at ISIS.

Atom	Site	*x*	*y*	*z*	Occ	B_{eq} (Å^2)
Sr1	2*c*	0.75	0.75	0.1917(2)	1	0.28(1)
Sr2	2*c*	0.75	0.75	0.4159(1)	1	0.32(1)
Fe1	2*c*	0.25	0.25	0.3141(1)	1	0.22(1)
Cu1	2*a*	0.25	0.75	0	1	0.45(2)
Se1	2*c*	0.25	0.25	0.0978(1)	1	0.39(1)
O1	4*f*	0.25	0.75	0.2934(1)	1	0.46(1)
O2	2*c*	0.25	0.25	0.4333(2)	1	0.39(1)

Table S4b. Refined atomic parameters from Rietveld refinement of Sr_2FeO_3CuS at 100 K using the PSD at I11.

Atom	Site	*x*	*y*	*z*	Occ	B_{eq} (Å^2)
Sr1	2*c*	0.75	0.75	0.18368(4)	1	0.05(7)
Sr2	2*c*	0.75	0.75	0.41167(4)	1	0.07(7)
Fe1	2*c*	0.25	0.25	0.30949(4)	1	0.05(1)
Cu1	2*a*	0.25	0.75	0	1	0.34(1)
S1	2*c*	0.25	0.25	0.0919(1)	1	0.25(2)
O1	4*f*	0.25	0.75	0.2898(2)	1	0.22(5)
O2	2*c*	0.25	0.25	0.4304(2)	1	0.11(6)

Table S4e. Refined atomic parameters from Rietveld refinement of Sr_2FeO_3CuS at 1.5 K using the WISH diffractometer at ISIS.

Atom	Site	*x*	*y*	*z*	Occ	B_{eq} (Å^2)
Sr1	2*c*	0.75	0.75	0.1835(2)	1	0.14(8)
Sr2	2*c*	0.75	0.75	0.4119(2)	1	0.38(7)
Fe1	2*c*	0.25	0.25	0.3094(2)	1	0.35(4)
Cu1	2*a*	0.25	0.75	0	1	0.54(6)
S1	2*c*	0.25	0.25	0.0917(5)	1	0.96(2)
O1	4*f*	0.25	0.75	0.2878(1)	1	0.71(5)
O2	2*c*	0.25	0.25	0.4315(2)	1	0.59(1)

References

(1) Brown, I. D.; Altermatt, D. Bond-valence Parameters Obtained from a Systematic Analysis of the Inorganic Crystal Structure Database. *Acta Crystallogr. Sect. B* **1985**, *41* (4), 244–247 https://doi.org/10.1107/S0108768185002063.
